# Molecular networking, conformal predictions and revised fingerprint-based models for discovering endocrine disruptors in mixtures

**DOI:** 10.1007/s00216-025-06303-2

**Published:** 2026-01-22

**Authors:** Yvonne Kreutzer, Ida Rahu, Ulf Norinder, Anneli Kruve

**Affiliations:** 1 Department of Chemistry, Stockholm University, Svante Arrhenius Väg 16, 106 91 Stockholm, Sweden; 2 Department of Environmental Science, Stockholm University, Svante Arrhenius Väg 8, 106 91 Stockholm, Sweden; 3 Department of Computer and Systems Sciences, Stockholm University, P.O.Box 1073, 164 25 Kista, Sweden; 4 MTM Research Centre, School of Science and Technology, Örebro University, 701 82 Örebro, Sweden

**Keywords:** Untargeted screening, Machine learning, Toxicity, Hazard, High-resolution mass spectrometry

## Abstract

Prioritizing high-risk features is a key step to reduce workload in non-targeted screening (NTS) when identifying environmental contaminants. Machine learning models from the MS2Tox toolbox have shown promise for feature prioritization, but rely heavily on the accuracy of molecular formulas and fingerprint features provided by SIRIUS + CSI:FingerID. In this study, we introduce and evaluate two new approaches—molecular networking (MN) and conformal predictions—to discover unidentified compounds potentially posing endocrine-disrupting activity based on tandem mass spectral similarity. Furthermore, we revised the previously published MS2Tox models, leveraging molecular fingerprints for seven Tox21 Data Challenge endpoints. The fingerprint-based MS2Tox models achieved the lowest false positive rate, 0.35, at 90% recall on the test set, while MN and CP yielded 0.82 and 0.68, respectively. In a case study of transformation products and persistent chemicals in wastewater, these three approaches prioritized 29 features in influent and effluent samples as potentially associated with AhR agonism among 189 LC/HRMS features corresponding to transformation products and persistent chemicals. All candidate structures for prioritized features showed scaffolds related to AhR binding affinity. Three features were identified on level 1, showcasing potential in using combined feature prioritization strategies.

**Graphical Abstract:**

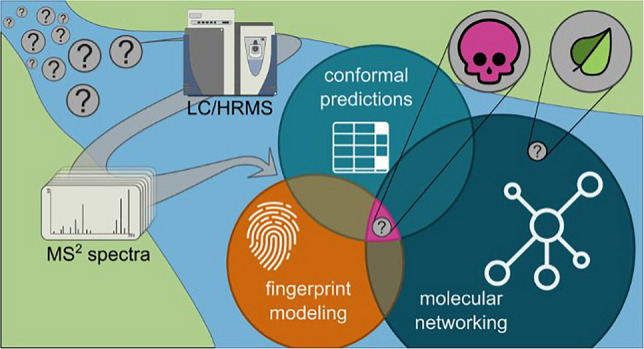

**Supplementary Information:**

The online version contains supplementary material available at 10.1007/s00216-025-06303-2.

## Introduction

Environmental water samples are highly complex mixtures containing hundreds to thousands of endogenous and exogenous compounds, with endocrine disruptors (EDs) posing one of the greatest concerns for both human health and ecosystems [1]. EDs have been linked to several major disorders; for instance, prenatal exposure to EDs has been associated with delayed cognitive and physical development in children, including lower birth weight and language delays [1].

Bioassays are frequently employed to assess endocrine-disrupting activity in environmental samples [2]; however, linking the measured activity from bioassays to individual compounds causing endocrine-disruptive effects is challenging and requires first identifying the detected compound, followed by in vivo, in vitro, or in silico toxicity assessment. Compound identification is commonly employed through analyzing a sample via non-targeted screening (NTS) with liquid chromatography (LC) or gas chromatography coupled to high-resolution mass spectrometry (HRMS) [3]. The detected features are interpreted by matching the MS^2^ spectra with experimental or in silico spectra from spectral libraries or predicting molecular fingerprints from MS^2^ spectra and then matching these with the ones from the structural databases [4]. Nevertheless, such structural elucidation is time-consuming and often produces multiple plausible structural candidates [5]. State-of-the-art workflows enable unequivocal identification of 0.1% to 10% of the LC/HRMS features, that is, the combination of exact mass and retention time, and possibly an MS^2^ spectrum [6]. For instance, Albergamo et al. [3] unequivocally structurally identified 15 and 10 compounds in positive and negative ionization modes, respectively, while 12,236 and 11,738 LC/HRMS features were detected in riverbank samples. Although NTS workflows are evolving to include toxicity assessments, the gap in measured and explained sample toxicity remains [7].

To streamline the feature identification process, it has been suggested to prioritize potentially toxic compounds before identification, based on analytical information, such as MS^2^ spectra, using machine learning (ML) models [8–15]. We recently introduced the MS2Tox toolbox, which includes models to predict the lethal concentration for 50% of the fish population (LC_50_) [8] and the biochemical activity in endpoints related to EDs [9] for compounds based on their MS^2^ spectra. In related work, Arturi et al. [10] developed MLinvitroTox, an ML tool pinpointing LC/HRMS features that are most likely to cause adverse effects, by predicting a toxicity fingerprint from MS^2^ spectra. These models use molecular fingerprints generated by SIRIUS + CSI:FingerID [16].

Limitations arise when fingerprint representations fail to capture relevant toxicophores, when fingerprints cannot be predicted from MS2 spectra, or when they are predicted inaccurately. The quality of the resulting fingerprint features depends strongly on the MS2 spectral quality, the assigned ion type, and the molecular formula. Uncertainties in any of these inputs can therefore lead to inaccuracies in the predicted fingerprints and, consequently, in downstream toxicity predictions [8]. In such cases, errors introduced at the fingerprint prediction stage propagate into the subsequent toxicity models, resulting in cumulative error and increased overall prediction uncertainty.

To mitigate this error propagation, approaches that predict compound properties directly from MS2 spectra and other experimental data, without an intermediate fingerprint prediction step, have been proposed [17, 18]. This motivates an investigation into whether toxicity-based LC/HRMS feature prioritization can be achieved using predictions derived directly from HRMS data.

A promising approach for gaining structural insights directly from MS^2^ spectra is through the use of molecular networking (MN) [19, 20]. Here, each MS^2^ spectrum is defined as a node, linked by edges representing a higher similarity than a predefined threshold. Thereby, compounds with similar MS^2^ spectra—and presumably similar structures—are closely linked to one another in the network. This principle has been successfully applied to annotate drug metabolites [23] and transformation products in water samples [24]. In related work, Zhao et al. [25] have developed an MN workflow that combines MS^2^ spectra with measured bioactivity for the fractionated sample, making it possible to assign higher priority to MS^2^ spectra, which are associated with higher bioactivity. The ability of MN to predict the biochemical activity of the unidentified features for discovering EDs is yet unexplored.

Furthermore, conformal predictions (CP) have shown promise in assessing the bioactivity prediction confidence of compounds using pre-defined error rates and have been shown to be especially useful for highly imbalanced datasets [26]. In previous work by Jin et al. [27], the CP framework was successfully applied to evaluate the uncertainty of predictions on the Tox21 dataset. However, CP is still to be applied to models predicting bioactivity directly from MS^2^ spectra.

In this work, we propose and evaluate three conceptually different frameworks to pinpoint LC/HRMS features related to EDs in environmental samples. We explore the possibility of pinpointing features related to seven nuclear receptor assays from the Tox21 Data Challenge [28] with MN, leveraging compounds with experimental MS^2^ data. We compare the performance of this approach with that of models that provide calibrated CP outcomes using MS^2^ data and models from the MS2Tox toolbox [9]. For this study, MS2Tox models were revised using binary fingerprints generated directly via the predefined *fingerprinter* function in SIRIUS, replacing the previous rcdk-based fingerprint calculation. Finally, all three approaches are applied to pinpoint EDs in a wastewater treatment plant influent and effluent samples.

## Materials and methods

### Data

**Fig. 1:**
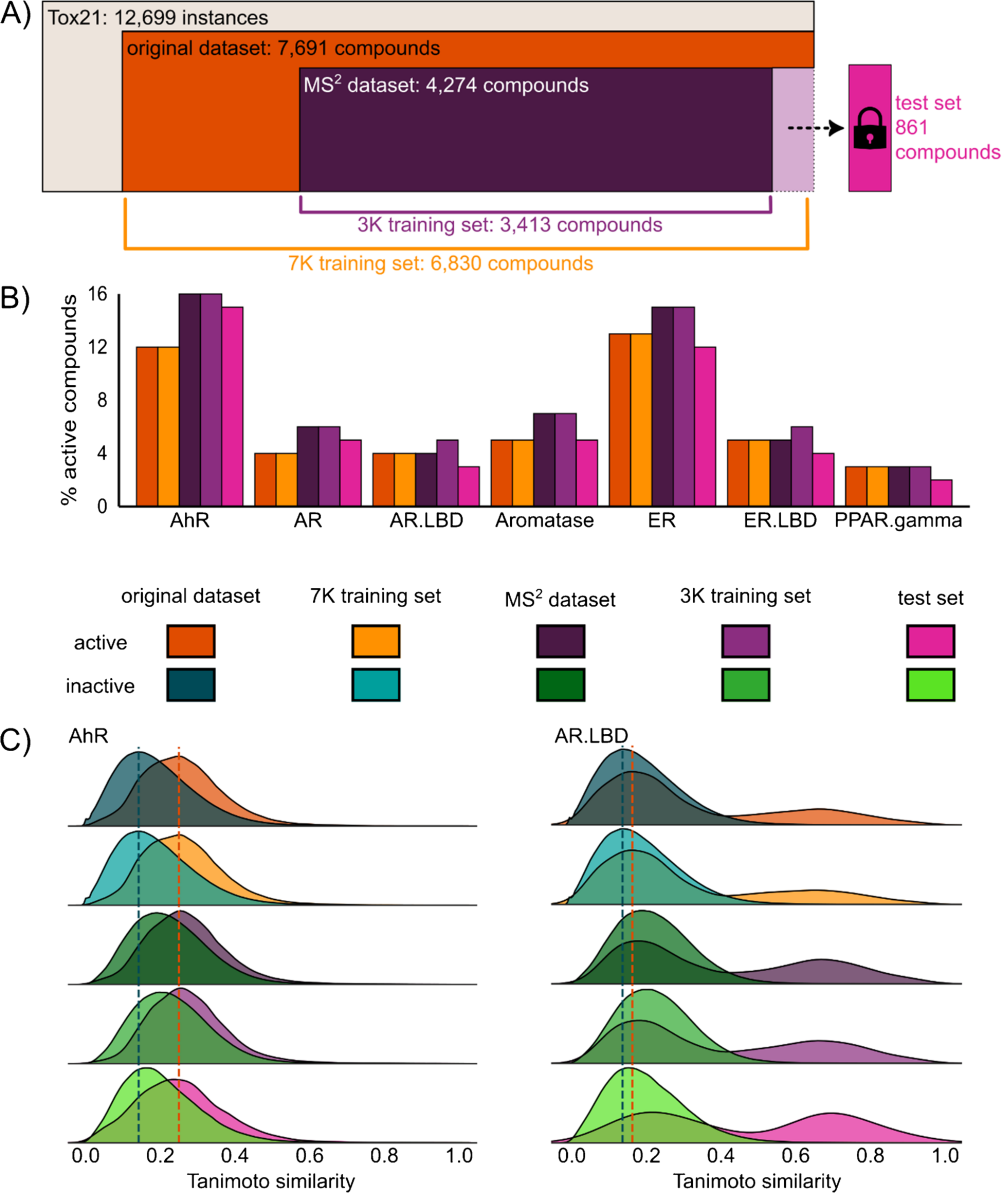
**A** Overview of datasets. **B** Percentage of active compounds per assay in each dataset. **C** Tanimoto similarity distribution of active-active and inactive-active compounds calculated from SIRIUS + CSI:FingerID general mode (3494 bits) binary fingerprints, for each dataset. The dotted lines represent the maximum of active-active (orange) and inactive-active (blue) compound similarity distributions in the original dataset. Remaining assays are provided in Fig. S2. The color legend encoding the different datasets is shared between **B** and **C**

The Tox21 Data Challenge dataset, containing 12,699 instances, was obtained from the official website (https://tripod.nih.gov/tox21/challenge/). The dataset was cleaned following a previously published data cleaning workflow [9], resulting in 7691 compounds with a unique first block (14 characters) of the InChIKey. Following the precautionary principle, a compound was deemed active in an assay if at least one active label was reported. Seven assays from the nuclear receptor panel were considered: activators of aryl hydrocarbon receptor (AhR), androgen receptor (AR), androgen receptor ligand-binding domain (AR.LBD), estrogen receptor (ER), estrogen receptor ligand-binding domain (ER.LBD), peroxisome proliferator-activated receptor gamma (PPAR.gamma), and inhibitors of aromatase (Aromatase). A brief description of these assay endpoints is provided in Table S1. The cleaned dataset will hereafter be referred to as the *original dataset* (Fig. 1A).

Due to the known scarcity of experimental HRMS data [13, 15], we undertook an extensive effort to compile the largest possible collection of MS^2^ spectra for compounds from the Tox21 dataset, using both public and commercial sources. Positive-mode LC/electrospray ionization (ESI)/HRMS spectra were gathered from MassBank Europe [29] (version 06.2024), MoNA [30] (version August 2024, downloaded 17.08.2024), GNPS (downloaded 25.07.2024), and NIST [31] (version HRMS 2023). Spectra were cleaned and averaged over all reported collision energies. For more detailed data processing information, see Text S1. Compounds from the *original dataset* with available MS^2^ spectra were selected to create the *MS*^*2*^* dataset* (4,274 unique compounds). This dataset was then split using an anticlustering algorithm [9, 32] into a *3 K training set* (80%, 3413 compounds) and a test set (20%, 861 compounds) to train the MS^2^ similarity-based approaches and MS2Tox-3K models. During the splitting process, it was ensured that the test set did not contain any compounds used for training SIRIUS + CSI:FingerID (v5.8.6). Additionally, a separate * 7 K training set*, comprising 6830 compounds disjoint from the test set, was compiled for training the MS2Tox-7K model. Incorporating the 7 K training set allows taking advantage of all available data, as MS2Tox models can be trained on absolute fingerprints calculated directly from chemical structures. Furthermore, it enables evaluation of the influence of data set size by comparison of the MS2Tox-3K and MS2Tox-7K models.

### Assay endpoint prediction

Compound activity in nuclear receptor signaling pathways was predicted for LC/HRMS features with three different approaches: MN, an unsupervised approach that assigns labels based on MS^2^ spectral similarity, here referred to as MN-MS^2^, and two supervised approaches: one that generates calibrated outcomes using CP from MS^2^ spectral similarity and MS2Tox that employs molecular fingerprints.

#### Molecular networking

The potential endocrine-disrupting activity of LC/HRMS features is predicted with MN-MS^2^ based on the activity labels (active as 1, inactive as 0) of directly connected nodes. Compounds marked as inconclusive in the original dataset were excluded from the analysis. Prediction performance was evaluated using a leave-one-out cross-validation approach. Parameters optimized for MN-MS^2^ construction included the type of spectral similarity metric, similarity threshold, edge filtering strategy, and voting scheme.

Three spectral similarity metrics were evaluated. Firstly, greedy cosine score, which represents the cosine of the angle of the vector representations of two spectra. Secondly, modified cosine score, which directly matches fragment ions and those shifted due to mass differences induced by modifications before calculating the cosine similarity [33]. The greedy cosine score and the modified cosine score were calculated with the functions *CosineGreedy()* and *ModifiedCosine()*, from the matchms [34] library in Python version 3.11.8, with a peak matching tolerance of 0.2 Da. Lastly, MS2DeepScore [35], an ML-based score, was calculated according to the published GitHub instructions [36]. MS2DeepScore utilizes a Siamese neural network, trained to predict the structural similarity, expressed through Tanimoto similarity of RDKit Daylight 2048-bit fingerprints, from the MS^2^ spectral pairs [35]. MS2DeepScore has been previously shown to outperform other metrics like the modified cosine similarity and Spec2Vec [35]. The similarity threshold, defining the minimum similarity required to form an edge between nodes, was optimized from 0.0 to 0.9 in 0.1 increments. Low similarity thresholds usually lead to large clusters of connected nodes; therefore, an additional edge filtering strategy was explored, where either the six most similar MS^2^ spectra were considered per node or all MS^2^ spectra with similarity scores above the threshold were included. Two voting schemes for calculating the probability of biochemical activity of a node were used: (i) majority voting, where the number of active connected nodes is divided by the number of connected active and inactive nodes (Eq. 1); and (ii) weighted voting, where the respective edge value is taken into account, therefore allowing more similar nodes to have a higher impact on the calculated probability of biochemical activity (Eq. 2).

1 $${p}_{\mathrm{majority}} = \frac{\text{number of active neighbors}}{\text{number of neighbors}}$$

2 $${p}_{\mathrm{weighted}}= \frac{\sum \text{similarity to active neighbors}}{\sum \text{similarity to all neighbours}}$$

Since initial experiments showed that the similarity threshold strongly affects the number of connected nodes, classical true and false positive rates were modified to include a penalty for overly high thresholds that leave many nodes unconnected. The modified true positive rate (mTPR) and modified false positive rate (mFPR) are defined as follows:

3 $$\mathrm{mTPR}= \frac{\mathrm{TP}}{\mathrm{TP}+\mathrm{FN}+\text{ NCP}}$$

4 $$\mathrm{mFPR}= \frac{\mathrm{FP}}{\mathrm{FP}+\mathrm{TN}+\mathrm{NCN}}$$

where TP is true positive, FN is false negative, FP is false positive, TN is true negative, and NCP and NCN are unconnected positives and unconnected negatives, respectively. Based on the mTPR and mFPR, the area under the modified receiver operating characteristic (mROC-AUC) was calculated and used for hyperparameter optimization. For each assay, the top three hyperparameter combinations, yielding the highest mROC-AUC, were identified. Overall, MN-MS^2^ leveraging MS2DeepScore combined with majority voting showed the best performance. The optimal similarity threshold was selected within this configuration.

The key assumption of using MN in NTS workflows is that structurally similar compounds exhibit similar biological activity. To evaluate this assumption, a reference network was constructed using the SIRIUS + CSI:FingerID general mode 3494-bit fingerprints calculated from SMILES representations of the compounds. Nodes in this network were connected based on their Tanimoto similarity. To clearly differentiate between approaches, the MS^2^ spectral similarity-based network is referred to as MN-MS^2^, and the fingerprint similarity-based network as MN–FP. Optimized MN-MS^2^ and MN–FP parameters are summarized in Table S2.

#### Conformal predictions

CP, developed by Vovk et al. [37, 38], is a mathematically grounded framework that guarantees a user-defined error rate, provided the data is exchangeable. CP operates as a post-processing step after model training, using an independent calibration set, which is typically a subset randomly selected from the training data prior to model training. In this study, we utilized the Mondrian version [38] of CP, which recalibrates predictions separately for each class in the dataset, ensuring the specified error rate is maintained. A random forest (RF) classifier was used as the underlying base model, and as features, MS2DeepScore similarities to all chemicals in the training set were employed. For more detailed information regarding the CP procedure, see Text S2.

#### Revising MS2Tox models for nuclear receptor assays

Model training was performed using Python (3.11.9), leveraging the XGBoost library to implement an XGBoost classifier. Hyperparameter optimization was performed using Optuna (v.3.6.1), with *RandomSampler()* on 500 trials and a fivefold stratified cross-validation (*StratifiedKFold* function from Scikit-learn) to preserve class distribution across folds. The optimized parameters and their selected values are provided in Table S3.

Two types of models were trained: MS2Tox-3K, using the * 3 K training set*, and MS2Tox-7K, using the larger * 7 K training set*. In contrast to our previous workflow, where fingerprint features were derived using the R package rcdk from SMILES strings, the current “reloaded” version employed a refined strategy. All SMILES were first standardized using the PubChem Power User Gateway (PUG) standardization service. Then, molecular fingerprints were computed directly using the standalone SIRIUS fingerprinter tool to ensure compatibility with the SIRIUS + CSI:FingerID (v5.8.6) format. To maintain consistency with prior work and improve robustness, only fingerprint features common to both positive and negative ESI modes were retained. Before training, feature cleaning was applied by removing zero- and near-zero-variance features and eliminating highly correlated fingerprint features to enhance model performance.

#### Approach comparison

The performances of the three presented approaches were compared based on the false positive rate at 90% recall (FPR_TPR=0.9_), as suggested by Rahu et al. [9], and the false positive rate at 50% recall (FPR_TPR=0.5_). These two metrics are well suited to capture the objective of toxicity-based feature prioritization. An effective model should pinpoint as many hazardous features as possible, corresponding to 50% and 90% of EDs, respectively, while prioritizing only a small number of features associated with inactive chemicals. The number of such inactive features directly determines the additional workload in an NTS workflow. Therefore, lower FPR_TPR=0.5_ and FPR_TPR=0.9_ values indicate higher prioritization efficiency, as fewer inactive features are prioritized, leading to a more effective reduction of workload.

### Wastewater analysis

Wastewater treatment plant influent and effluent samples were stored at −20 °C, filtered through a 0.45-µm pore size syringe filter, and subsequently mixed with methanol to yield a methanol concentration of 20%. The samples and a blank were analyzed with LC/ESI/HRMS. A reversed-phase C18 chromatographic column with a gradient elution, facilitating a 0.1% formic acid water and acetonitrile as mobile phase components, was used for separation. The MS^1^ and MS^2^ spectra were acquired with a Q Exactive Orbitrap (Thermo Fisher Scientific, USA) in positive mode. Data-dependent acquisition was used to trigger MS^2^ with an inclusion of 176 relevant water contaminants from the NORMAN suspect list [39] (Table S4) combined with top5. All experimental details on the analysis of wastewater samples are presented in Text S3.

Samples were analyzed in duplicates. The resulting LC/HRMS features were processed with MS-DIAL (version 5.3.240719, parameters in Table S5), and AhR activity was predicted with all three approaches. LC/HRMS features were further prioritized to pinpoint potentially persistent compounds (feature intensity change within ± 20% between influent and effluent) or transformation products (at least 50% higher intensity in the effluent compared to the influent). Candidate structures were retrieved with SIRIUS + CSI:FingerID (v5.8.6) and MassBank library (version 2024.11) search. Analytical standards were obtained for zolpidem phenyl-4-carboxylic acid, carbamazepine, and carbamazepine 10,11-epoxide and used to evaluate the tentative structural annotation in addition to AhR activity testing. Further experimental details, alongside data processing and in vitro AhR assay procedure, are given in Text S4.

## Results and discussion

### Data overview

The *MS*^*2*^* dataset* (4274) captures the majority of the chemical space of the original dataset (7691) (Fig. S1). Despite extensive efforts to assemble the largest possible collection of MS^2^ data for chemicals included in the Tox21 Data Challenge, certain regions of chemical space remain underrepresented. The proportion of active compounds in the *MS*^*2*^* dataset* varies from 3% (PPAR.gamma) to 16% (AhR), and closely aligns with the proportions observed in *the original dataset* (Fig. 1B)*.*The number of active compounds in the held-out test set ranges from 17 in PPAR.gamma up to 108 in the AhR (Table S6). This is attributed to the overall low proportion of active compounds in the Tox21 dataset, combined with the restriction to compounds for which MS^2^ spectra were available and which were not part of the SIRIUS + CSI:FingerID training set. The limited number of active compounds poses challenges for statistically robust evaluation, particularly for assays with a low proportion of active compounds.

To further assess the representativeness of the datasets, pairwise Tanimoto similarities were calculated between all the active compounds and all other compounds (active-active and active-inactive) within the datasets (Fig. 1C). For instance, in the case of AhR, the similarity distribution of active-active and inactive-active compounds was consistent across datasets; the maximum of active-active similarity distribution in the *original dataset* aligns with the distribution maximum across all datasets. For AR.LBD, a distinct cluster of highly similar active compounds was observed. This cluster corresponds to structurally highly similar steroids and steroid derivatives, which are characterized by fused four-membered ring systems and are well-known AR and AR.LBD agonists [40]. Nevertheless, for AR.LBD, the active-active similarity distribution does not align across datasets, indicating that the subsets contain more structurally similar active compounds compared to the original dataset. In particular, the *test set* shows a higher proportion of similar compounds, reflected by an increased proportion of compounds with Tanimoto similarity between 0.6 and 0.8 compared to the *original dataset* (Fig. 1C). This can also be observed for AR.LBD, ER, ER.LBD, and PPAR.gamma (Fig. S2); therefore, while the models were trained for all assays, a fair comparison is feasible only for AhR and AR, where the test set is representative of the * 3 K training set*.

### Molecular network parameter selection and optimization results

**Fig. 2:**
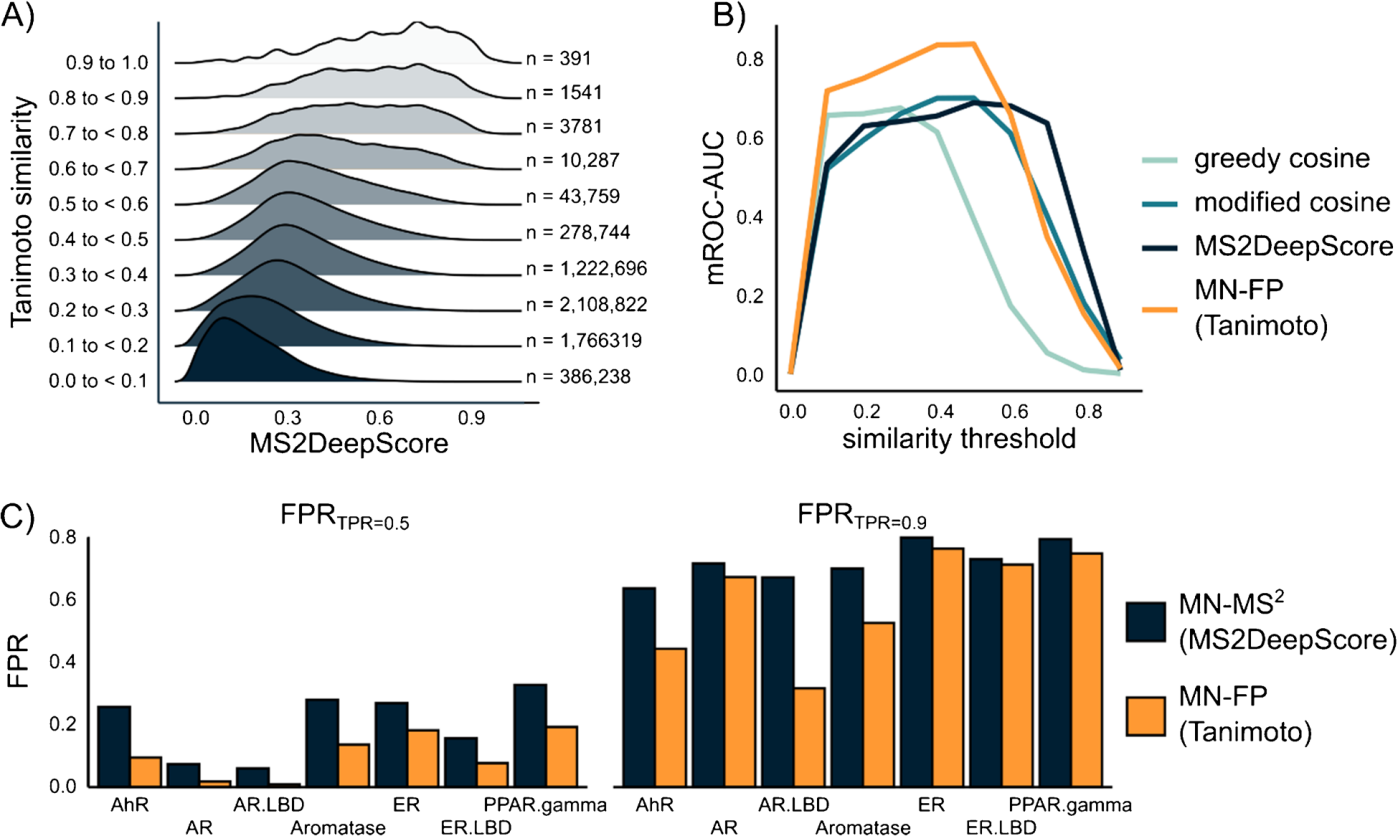
**A** Correlation between pairwise structural Tanimoto similarity and pairwise MS^2^ similarity (MS2DeepScore) of the same compound. **B** mROC-AUC values depending on the similarity threshold for all the tested MN parameters for the AhR assay. **C** Obtained FPR_TPR=0.5_ and FPR_TPR=0.9_ for MN-MS^2^ and MN-FP at optimized hyperparameter settings

The MS^2^ spectra of 3413 compounds in the * 3 K training set* yielded 5,822,578 pairwise similarity values for each similarity metric, with most values falling below 0.5. The modified cosine similarity and the MS2DeepScore exhibited similar distributions, while the greedy cosine similarity distribution was skewed towards lower values (Fig. S3). The calculated Spearman correlation coefficient between spectral similarity and structure-based Tanimoto similarity was low across all metrics due to the skewed distribution; however, MS2DeepScore achieved the highest *R*^2^ of 0.38 (Table S7). Still, pairs of compounds with high spectral similarity tended to have high Tanimoto similarity, whereas high Tanimoto similarity could also exhibit low spectral similarity across metrics. This suggests that while high spectral similarity can imply structural similarity, the reverse does not necessarily hold true (Fig. 2A). One explanation is that some MS^2^ spectra may contain limited structural information compared to fingerprints calculated from SMILES, e.g., due to lower spectral quality.

MN hyperparameters — including the type of similarity metric, similarity threshold, voting scheme, and edge filtering strategy — were optimized based on mROC-AUC. Overall, the performance remained consistent regardless of the voting scheme and was slightly influenced by the type of similarity metric (Fig. 2B, Fig. S4). Nevertheless, a comparison of the three best-performing hyperparameter combinations across assays showed a slight preference for MS2DeepScore, which was therefore selected as the default similarity metric for MN-MS^2^ predictions. In addition, MN-MS^2^ was evaluated both with edge filtering (retaining the six most similar edges per node) and without limiting the number of edges. Results indicated that edge filtering is beneficial at very low similarity thresholds (£0.2); but overall, higher mROC-AUC values were obtained when all edges above the threshold were retained (Fig. S5). The best-performing similarity threshold varied by assay: 0.3 for Aromatase, 0.4 for AR.LBD, 0.5 for AhR, ER.LBD, and PPAR.gamma, and 0.6 for AR and ER.

The performances of MN-MS^2^ and the MN-FP for prioritization of LC/HRMS features in NTS workflows were compared based on FPR_TPR=0.5_ and FPR_TPR=0.9_. The FPR_TPR=0.5_ was lowest for AR.LBD, with values of 0.06 for MN-MS^2^ and 0.01 for MN-FP, likely due to a cluster of highly similar active compounds (Fig. 2C). Across all assays, MN-FP showed consistently lower FPR_TPR=0.5_ compared to MN-MS^2^, likely because molecular fingerprints more closely represent structural similarity than MS^2^ spectra. The FPR_TPR=0.9_ was high across all assays for the MN-MS^2^, ranging from 0.67 for AhR to 0.84 for ER (Fig. 2C, Table S2). The notable increase from FPR_TPR=0.5_ to FPR_TPR=0.9_ suggests that identifying the first 50% of active compounds is relatively straightforward, whereas the subsequent 40% exhibit greater similarity to inactive compounds, making them more challenging to distinguish.

While a key advantage of using MN lies in the visualization of complex non-targeted MS datasets and the identification of previously unreported compounds, this strength is most pronounced in structurally homogeneous compound classes [41, 42]. Compounds posing endocrine-disrupting activity are known for complex underlying mechanisms of action, yielding a wide range of compounds showing similar endocrine-disruptive effects [43, 44]. Thus, assessing the endocrine-disrupting activity of unidentified features solely on their MS^2^ similarity is insufficient for capturing key scaffolds, attested by the high FPR_TPR=0.9_ of MN-MS^2^ and MN–FP. 

### Conformal predictions

CP operates on a transformed feature space (reliability domain) and remains unaffected by the unequal distribution of active compounds in different datasets. A transformed feature space is obtained through the underlying algorithm, in this case, RF, in combination with the calibration set [45]. The CP predictions on the test sets show that, for the most part, the datasets have error rates in agreement with the set error limits (Fig. S6); however, the active class of the ER.LBD test set consistently yielded an error rate exceeding the acceptable limit for all levels of investigated error rates. Furthermore, the efficiency of the models, i.e., providing a single label (active or inactive) prediction, is rather low for significance levels below 0.25 and far below 80% in many cases (Fig. S6). At a significance level of 0.30, most of the models have acceptable efficiencies. The overall low efficiencies at significance levels 0.15 and 0.20 for most assays indicate that information in the MS^2^ spectral similarity is insufficient for activity predictions and that additional information is needed to achieve the aforementioned error rates alongside higher predictive capabilities in terms of efficiency. Detailed test set performances are provided in Table S9.

### Comparative evaluation of prediction approaches using the test set

The high FPR_TPR=0.9_ observed for both MN-MS^2^ and MN-FP underscores the inadequacy of relying solely on an unsupervised approach utilizing spectral or structural similarity for annotating compounds potentially posing endocrine-disrupting activity. To investigate this further, the performance of the MN-MS^2^ approach was compared with supervised approaches, namely CP, trained on pairwise MS2DeepScore spectral similarity, and MS2Tox models, trained on SIRIUS + CSI:FingerID fingerprints. Given the previously discussed limitations of the test set, the following analysis focuses on the AhR assay. For this assay, 108 active compounds were included in the test set, where active-active and inactive-active similarity distributions align with the ones from the * 3 K training set*, and exchangeability at all significance levels for CP prediction is guaranteed. Test set results for all approaches and assays can be found in Tables S8 – S12.

**Fig. 3:**
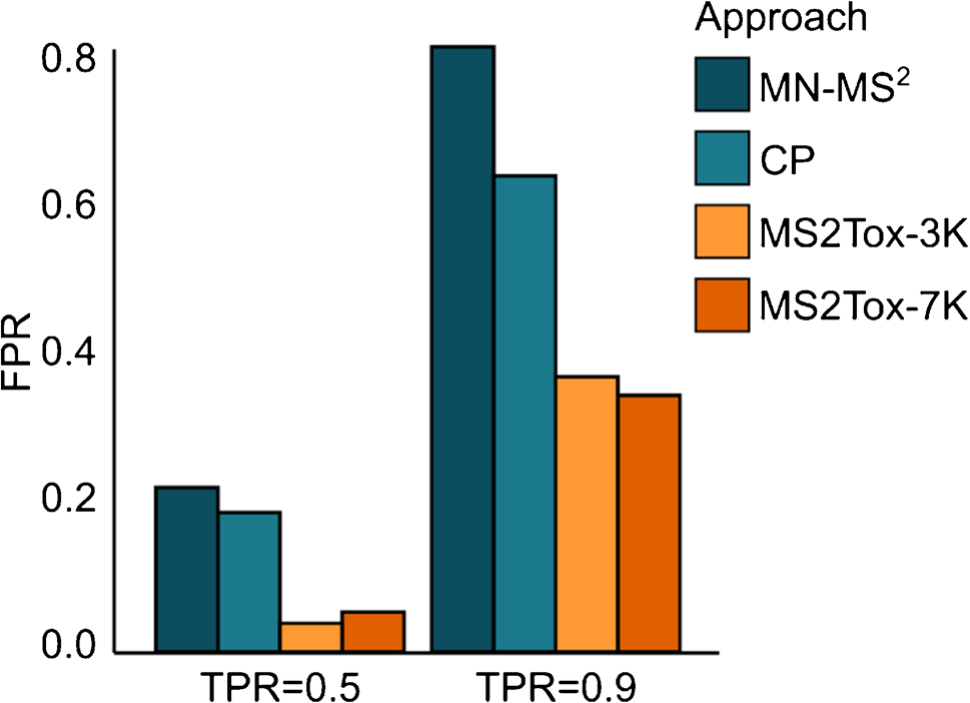
Comparison of FPR_TPR=0.5_ and FPR_TPR=0.9_ between approaches for AhR on the test set (*n* = 861, number of actives in the test set is 108 for AhR)

The FPR_TPR=0.5_ for MN-MS^2^ and CP resulted in 0.22 and 0.19, respectively. At threshold TPR = 0.9, the FPR values increased for both approaches, though remaining lower for CP (0.82 for MN-MS^2^ and 0.68 for CP). In contrast, the FPR_TPR=0.5_ and FPR_TPR=0.9_ for the MS2Tox-3K approach were substantially lower, 0.04 and 0.38, respectively (Fig. 3, Fig. S7). This is likely due to MS^2^-based approaches inadequately capturing structural features relevant to AhR activity.

The active compounds correctly predicted by the approaches overlapped at threshold TPR = 0.9; however, differences were observed at threshold TPR = 0.5. At this threshold, approaches correctly identified the same 22 active compounds, while 14 additional compounds were exclusively identified by fingerprint-based MS2Tox models, and 12 were correctly identified only by MS^2^ similarity-based approaches (MN-MS^2^ and CP). This suggests that the different approaches may capture complementary aspects of the data, though no consistent structural characteristics were found among the compounds uniquely identified by each approach (Fig. S8). Fingerprint-based models are independent of the availability of MS^2^ spectra, as they are trained on fingerprints derived directly from molecular structures. Thus, additional models, namely MS2Tox-7K models, were trained on the larger * 7 K training set*. On the test set, MS2Tox-7K models achieved similar FPR_TPR=0.5_ and FPR_TPR=0.9_ values compared to the MS2Tox-3K models (Fig. 3, Tables S10–S12). Nevertheless, the performance gap between the MN-MS^2^ and CP approaches compared to the MS2Tox-3K and MS2Tox-7K approaches is higher than within MS2Tox-3K and MS2Tox-7K, indicating that the type of input features (MS^2^ spectral similarity vs fingerprint features) is a more influential factor than the training set size.

### Case study: wastewater analysis

The MN-MS^2^ and CP models were re-optimized and retrained on the *MS*^*2*^* dataset*, and the fingerprint-based model was retrained on the original dataset prior to application for prioritization of LC/HRMS features detected in wastewater samples. In all cases, the prediction thresholds were set to achieve 90% recall.

From the measured influent and effluent wastewater samples, a total of 4375 features with unique precursor *m/z* and retention time were extracted with MS-DIAL after componentization and blank subtraction. About one-quarter (968) of these had associated MS^2^ spectra containing five or more peaks with relative intensity above 5%. Features were further prioritized to pinpoint potentially persistent compounds or transformation products by considering the change in intensity between influent and effluent (section “Wastewater analysis”). Among the 968 features, 40 were deemed to correspond to potentially persistent compounds based on their feature intensity change remaining within ±20% between influent and effluent, and 153 were classified as potential transformation products based on at least a 50% higher intensity in the effluent compared to the influent. This resulted in a total of 193 features of interest.

**Fig. 4:**
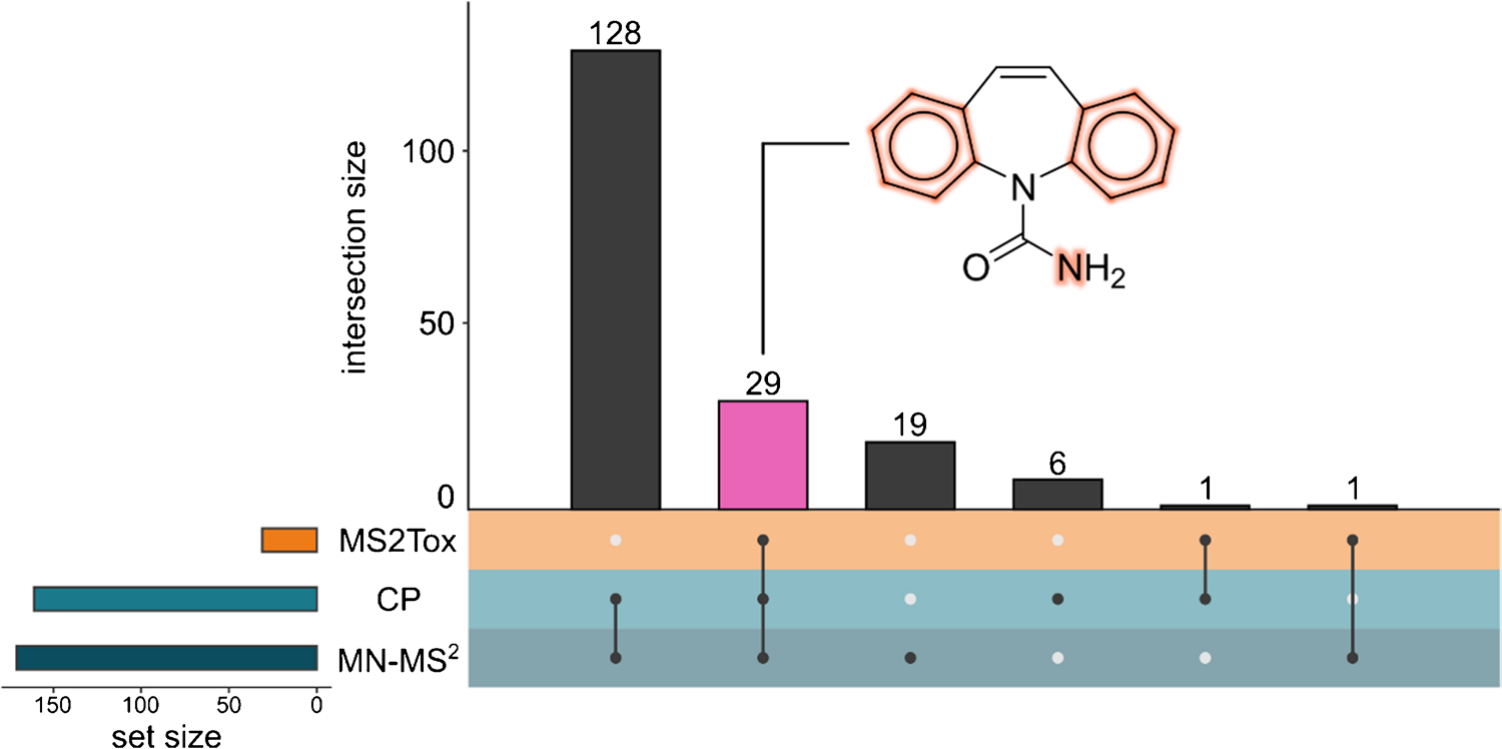
UpSet plot of the labeling results for the 193 LC/HRMS features of interest, with 29 features highlighted that were labeled by all three approaches. CP and MN-MS^2^ parameters were retrained and re-optimized with the MS^2^ dataset. MS2Tox refers to the fingerprint-based model retrained on the original dataset. The structure of carbamazepine, falsely predicted as active by all approaches, is shown. Moieties, corresponding to the three most important fingerprint features according to SHAP analysis, are highlighted

All three approaches were applied to predict the biochemical activity with respect to potential AhR agonism of these features. Both the MN-MS^2^ and CP models predicted 128 features as active, while all three approaches agreed on 29 features as potential AhR agonists (Fig. 4). Nine features were labeled as inactive by all of the approaches. For the 29 commonly prioritized features, candidate structures were retrieved using four different methods: (1) library matching with MassBank using a cosine similarity threshold of 0.7 and requiring at least four matching fragment peaks; (2) querying the compound from the *MS*^*2*^* dataset* (MassBank, MoNA, NIST overlap with Tox21) with the highest MS2DeepScore for the detected LC/HRMS feature; (3) retrieving the top-ranked candidate structure from SIRIUS + CSI:FingerID; and (4) retrieving the top-ranked candidate structure for the highest ranked molecular formula in SIRIUS + CSI:FingerID. Candidate structures were obtained for all 29 prioritized LC/HRMS features using methods 2 and 3, for 27 features using method 4, and for two features using method 1 (Table 1, all candidates are shown in Fig. S10). This resulted in a total of 87 candidate structures.

**Table 1:**
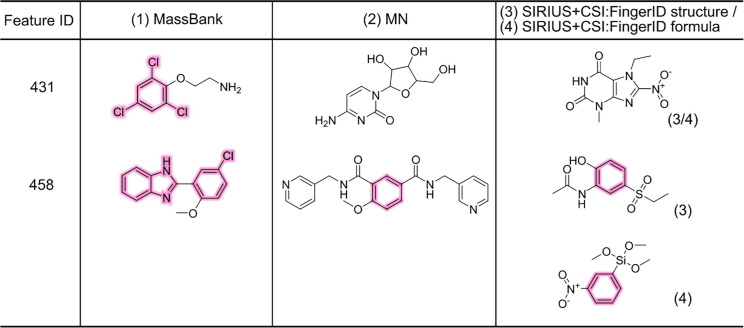
Potential candidate structures for two selected LC/HRMS features (candidates for the remaining features are provided in SI4)

Candidate structures were examined to detect structural patterns that were previously reported to be associated with AhR binding affinity [46–52]. Of the 87 candidate structures, 65 contained at least one relevant structural pattern, namely an aromatic ring (benzene). Furthermore, eight of these featured a halogenated aromatic ring. Additionally, one candidate structure contained an indole moiety, two contained a biphenyl structural pattern, and three contained benzimidazole scaffolds. Dioxin-like compounds, known for their strong AhR activity and environmental persistence, are typically expected in wastewater samples. However, none was identified among the candidate structures in this study. This may reflect either their true absence in the samples or a limitation of the analytical approach, as LC was used here, whereas dioxin detection typically relies on gas chromatography [53].

Candidate structures retrieved from SIRIUS + CSI:FingerID should be interpreted with caution, as candidate structure retrieval and activity predictions with fingerprint-based MS2Tox models rely on the same fingerprints generated with SIRIUS + CSI:FingerID. This assumption presumes the accuracy of the SIRIUS + CSI:FingerID fingerprints, which could introduce potential bias in the activity predictions.

To further prioritize candidates, their bioactivity was cross-referenced with existing annotations in the Tox21 dataset. Two candidates retrieved via method 2 were annotated as active. In contrast, only one candidate (carbamazepine) retrieved via methods 3 and 4 was present in the Tox21 dataset but annotated as inactive. No candidate structures from the MassBank library matching were found in the Tox21 dataset. This limited overlap highlights the need to expand current toxicological datasets to improve risk assessment capabilities.

Three structural candidates were confirmed with analytical standards, achieving a confidence level of 1 according to the Schymanski scale [4]. Among them, carbamazepine and its transformation product, carbamazepine 10,11-epoxide, were labeled as active by MS2Tox, CP, and MN-MS^2^. Their presence was confirmed by matching MS^2^ spectra and retention time with analytical standards (Fig. S10). Although carbamazepine was labeled as active by all three approaches, the compound is annotated as inactive in Tox21, making this prediction a false positive. Carbamazepine and carbamazepine 10,11-epoxide were also experimentally tested with effect-based analysis regarding AhR activity, performed as described in Lundqvist et al. [54]. Both compounds were found to be inactive. Nevertheless, in a recent publication by Kanonjia et al. [55], carbamazepine has been found to be a weak AhR agonist. Furthermore, carbamazepine has been previously reported as a persistent compound [56], and carbamazepine 10,11-epoxide is its transformation product. This highlights the added value of combining multiple complementary prioritization approaches.

The false positive labeling of carbamazepine by the MS2Tox model was attempted through feature importance with SHAP analysis (Table S13 and Fig. S11). The most important fingerprint features from MS2Tox correspond to whether a structure contains two or more aromatic rings, a six-membered aromatic ring composed exclusively of carbon atoms, and a nitrogen atom connected to at least one hydrogen atom and to a carbon atom, which all contribute towards increasing the probability of an LC/HRMS feature being predicted as active. All these moieties are present in carbamazepine, and it can therefore be explained why it was falsely predicted as active (Fig. 4). The false positive labeling from MS^2^ similarity approaches can be explained through the investigation of connections with the highest MS2DeepScore. For MN, in total, 171 compound database spectra were connected to the LC/HRMS feature corresponding to carbamazepine, out of which 39 (22.8%) were active. To achieve a recall of 90%, the minimum probability of an LC/HRMS feature being active was set to 0.14, and the probability of the feature exceeded this threshold. This highlights the expected effect of low similarity thresholds (here 0.5): features are prioritized even if a high fraction of similar features is inactive. This furthermore feeds into the general observed high false positive rates seen through the test set. Features predicted as active solely by MN-MS^2^ and CP included zolpidem phenyl-4-carboxylic acid (level 1 identification, Fig. S12), which was experimentally deemed inactive for AhR, showcasing the high false positive labeling by MS^2^-similarity approaches. Additionally, SIRIUS + CSI:FingerID fingerprints could not be predicted for 25 features predicted to be active by MN-MS^2^ and CP. Hence, MS2Tox model predictions remained inaccessible. One of these features matched chloridazon in MassBank (0.7 similarity, 4 matched fragments), but was confirmed to be a wrong candidate structure with the reference standard. All in all, MS^2^ similarity-based approaches are independent of the in silico MS^2^ interpretation tools, such as SIRIUS + CSI:FingerID, but show a high false positive rate, thereby limiting the prioritization capability.

## Conclusion

In this study, we explored the potential of MN, CP, and fingerprint-based ML models to discover LC/HRMS features potentially associated with endocrine-disrupting activity prior to their structural elucidation. Comparing MS^2^ spectral similarity-based approaches (MN-MS^2^ and CP) and fingerprint-based MS2Tox models demonstrated that spectral similarity alone is insufficient for reliably predicting bioactivity, by exhibiting higher false positive rates compared to fingerprint-based MS2Tox models. Furthermore, the performance comparison emphasizes the advantage of incorporating supervised ML approaches and more informative training data in the form of molecular fingerprints instead of solely spectral similarity. We also demonstrated the application of all three prioritization approaches using influent and effluent samples, where retrieved candidate structures of prioritized features contained structural scaffolds associated with AhR interaction. Two features, predicted active by all approaches, could be identified on level 1, which are potentially connected to AhR agonism. While we demonstrate that prioritizing features based on more than one property is beneficial, this study highlights the need for extending spectral databases and reliable toxicity data labels to enable advances in ML-assisted feature prioritization approaches in NTS workflows for complex mixtures.

## Supplementary Information

Below is the link to the electronic supplementary material.

Supplementary Material 1 (PDF 3.06 MB)

## Data Availability

The code and fingerprint models are available from GitHub https://github.com/kruvelab/MS2Tox/tree/main/MS2Tox_molecular_networking, and the raw data and MS-DIAL summary files for the wastewater analysis are available upon request. Sharing the full training data violates the NIST-23 license agreement; therefore, only a subset can be shared.
