# Supplementary material for: Molecular networking, conformal predictions and revised fingerprint-based models for discovering endocrine disruptors in mixtures
Source: Anal Bioanal Chem. 2026 Jan 22;418(5):1445–57. doi: 10.1007/s00216-025-06303-2 (PMC12909378; doi:10.1007/s00216-025-06303-2)
Supplement: Supplementary file 1 — Supplementary Material 1 (PDF 3.06 MB) [file 216_2025_6303_MOESM1_ESM.pdf]

Supporting Information:

Molecular Networking, Conformal Predictions and Revised Fingerprint-based Models for Discovering Endocrine Disruptors in Mixtures

*Yvonne Kreutzer,[a] Ida Rahu,[a,b] Ulf Norinder,[c,d] Anneli Kruve[a,b]**

[a] Department of Chemistry, Stockholm University, Svante Arrhenius väg 16, 106 91 Stockholm, Sweden

[b] Department of Environmental Science, Stockholm University, Svante Arrhenius väg 8, 106 91 Stockholm, Sweden

[c] Department of Computer and Systems Sciences, Stockholm University, P.O.Box 1073, SE-164 25 Kista, Sweden

[d] MTM Research Centre, School of Science and Technology, Örebro University, 701 82 Örebro, Sweden

**Corresponding author: anneli.kruve@su.se*

Table of Contents

Text S1. Data processing 3
Text S2. Conformal Predictions 4
Text S3. Extended information on Wastewater sample analysis 5
Text S4. AhR testing protocol 6

Table S1. Description of nuclear receptor assays. 7
Table S2. Comparison of MN-MS^2 based on MS2DeepScore and MN-FP 8
Table S3. Selected hyperparameters and tried values of the XGBoost MS2Tox models. 9
Table S4. Inclusion list of environmentally relevant contaminants 10
Table S5. Applied MS-DIAL parameters 14
Table S6. Number of available compound labels for each assay in the test set (N = 861). 16
Table S7. Spearman correlation 17
Table S8. MN-MS^2 test set results. 18
Table S9. CP test results. Bold rows indicate the best significance and CoverageP per endpoint. 19
Table S10. MS2Tox models test set performance without constraints. 21
Table S11. MS2Tox models test set performance at threshold resulting in TPR=0.5. 22
Table S12. MS2Tox models test set performance at threshold resulting in TPR=0.9. 23
Table S13. SHAP analysis of the MS2Tox AhR model 24

Fig. S1 Chemical space visualization with UMAP 25
Fig. S2 Tanimoto similarity distribution 26
Fig. S3 Distribution of calculated similarities between spectra pairs of each calculated similarity type 27
Fig. S4 mROC-AUC values for MN activity annotation for all hyperparameter combinations of all bioassay endpoints 28
Fig. S5 mROC-AUC values for activity annotation on MS2DeepScore MN-MS^2 29
Fig. S6 Efficiencies of the investigated test data sets at various significance levels 30
Fig. S7 Comparison of FPR at 50% recall and at 90% recall 31
Fig. S8 UpSet plot of overlapping true positive predictions in the test set for AhR at 50% recall 32
Fig. S9 Candidate structures. Scaffolds associated to potential endocrine disruptive activity are highlighted. 37
Fig. S10 Carbamazepine and carbamazepine 10,11-epoxide spectra comparison to spectral references. 38
Fig. S11 Variable importance analysis of the MS2Tox AhR activity prediction model 39
Fig. S12 Confirmation of Zolpidem phenyl-4-carboxylic acid 40

Text S1. Data processing

All spectra with a precursor ion mass below 1000 Da and ion type $[M+H]^+$ were considered. For compounds (based on the first 14 characters of InChIKey, later denoted as InChIKey14) with spectra available in all three databases, the respective spectra were taken from NIST, as NIST incorporates spectra recorded over a wide range of collision voltages for all compounds and are therefore considered more informative. Similarly, MassBank spectra were preferred over MoNA spectra and MoNA spectra over GNPS spectra. To avoid overrepresentation of more frequently studied chemicals, the spectra, corresponding to the same compound (unique InChIKey14) measured with different collision energies, were averaged. Peaks with a relative intensity below 5% were classified as noise and removed. The *m/z* of all detected ions in MS^2 spectra were binned with one decimal and intensities were scaled between 0 and 1. In case *m/z* of several peaks fell in the same bin, the highest intensity one was selected. Furthermore, only spectra with 5 to 1000 fragment peaks were considered. In total, spectra of 4274 compounds with unique InChIKey14 were retrieved based on their presence in the Tox21 data set. For data set splitting the compounds that were used to train SIRIUS+CSI:FingerID were destined to be in the training set. The remaining compounds were grouped by using anticluster splitting.[1] The *anticlustering()* function from the R package *anticlust*[2] with k = 100 was used for generating 100 clusters and 51 of the clusters were randomly selected as the test set. The remaining 80%, 3413 compounds with respective spectra, were used for training.

Uniform Manifold Approximation and Projection (UMAP)[3] by using the Python library umap with the *UMAP()* function.

Text S2. Conformal Predictions

Scikit-learn[4] (version 0.23.2) was used for building the underlying random forest models (RandomForestClassifier) and nonconformist (version 1.2.5, https://github.com/donlnz/nonconformist) for building the Mondrian conformal predictors. Default parameters were used. Ten models were built with a, randomly selected (seed=1234), calibration set of 30 % from the training set leaving 70 % for the actual training of the RF model. The 10 models were subsequently used to predict the corresponding test set as well as the wastewater spectra. Median conformal p-values from the ten models were used for each predicted spectra and class, respectively, as the outcome of the analysis.

The Conformal Predictor (CP) output for binary classification is one of 4 possibilities: A single label prediction for either of the 2 classes, both labels (both classes) or no label (empty class). Important outcome parameters for quantifying the quality of the derived CP models are validity and efficiency.

Validity (for each class for Mondrian conformal predictors) is the percentage of correct predictions at the user-set significance level (a user set percentage of acceptable errors). Here it should be noted that the "both" prediction is always correct for binary classification as it always contains the correct class while the empty prediction is always erroneous since it contains no classes.

Efficiency (for each class for Mondrian conformal predictors) is the percentage of single-label class predictions regardless of whether these predictions are correct or not.

For a more detailed description of the CP procedure, see reference Norinder et al.[5]

Thus, the aim is therefore as high, informative, percentage single label class predictions as possible, i.e. high efficiency, at the user-set significance level in question.

The MS^2 spectra described above were used as input for feature generation. For every test set and wastewater set spectrum a similarity analysis was performed versus each of the training set spectra using the MS2Deep score. This generated a feature similarity matrix with number of columns equal to the number of training set spectra and rows equal to the number of test set or wastewater spectra, respectively. To mitigate the large similarity values (1.0) along the diagonal compared to other values in the training set data matrix the diagonal values were reduced to 0.5.

Text S3. Extended information on Wastewater sample analysis

Wastewater treatment plant influent and effluent samples from Sweden were stored at –20 °C, prior to analyses the samples were filtered through a 0.45 µm pore size syringe filter and 20% of methanol was added. Samples were analysed on the LC system Dionex UltimateTM 3000 UHPLC with an RS pump, RS autosampler, and RS column compartment (Thermo Fischer Scientific, USA). The column for chromatographic separation was a C18 reversed-phase column Kinetex (Phenomenex, Germany) with dimensions of 150 x 3.0 mm and a particle size of 2.6 µm. The column was kept at 40 °C. The mobile phase consisted of 0.1% formic acid (VWR Chemicals, France) in HPLC grade water (Honeywell, Germany) and 0.1% formic acid in acetonitrile (Honeywell, Germany) and HPLC grade water (95:5). The gradient started with 5% organic phase and linearly increased to 100% with 20.0 min, was held constant for 5 min and thereafter lowered back to 5% organic phase with 0.1 min. The equilibration time between injections was 4.9 min.

HRMS analysis was directly coupled to the LC system and performed on a Q Exactive Orbitrap (Thermo Fisher Scientific, USA) with electrospray ionization (ESI). All measured samples were acquired in data-dependent acquisition (DDA) in ESI positive mode. The ESI spray voltage was 3.5 kV, the capillary temperature was 320 °C, the probe heater temperature was 320 °C, and the maximum spray current was 100 µA. S-lens RF level was set to 50% and the gas parameters were set to 50 arbitrary units (AU) for the sheath gas, 3.0 AU for aux gas, and 0.0 AU for spare gas. The LC/HRMS features with corresponding MS^2 spectra were extracted with MS-DIAL (version 5.3.240719). Detailed parameter settings can be found in Table S5. The features were further processed with the three different approaches to labeling spectra related to endocrine activity.

Text S4. AhR testing protocol

An effect-based method was used to analyze the AhR activity of a pure compound. The effect-based analysis was performed as described in Lundqvist et al.[6]

In general, compounds are analyzed in dilution series, and they are classified as AhR active if they have an activity >10% of assay maximum (defined by a concentration of TCDD that is saturating the assay). The potency of compounds is then expressed as the effect-concentration 10% (EC10), i.e., the concentration of the compound needed to increase the activity in the assay to 10% of assay maximum.

In this project, the following compound was tested in a dilution series with the highest exposure concentration as listed below:

Carbamazepine (Sigma-Aldrich, China): 500 μM

Carbamazepine 10,11-epoxide (Sigma-Aldrich, Germany): 500 μM

Zolpidem phenyl-4-carboxylic acid (Sigma-Aldrich, Germany): 500 μM

The tested compounds did not exert any AhR activity in the tested concentration range. Raw data can be provided upon request.

Table S1. Description of nuclear receptor assays

Assay	Description
Ahr	Activation of the aryl hydrocarbon receptor
AR	Activation of the androgen receptor
AR.LBD	Binding to the ligand-binding domain of the androgen receptor
Aromatase	Inhibition of aromatase activity
ER	Activation of the estrogen receptor
ER.LBD	Binding to the ligand-binding domain of the estrogen receptor
PPAR.gamma	Peroxisome Proliferator-Activated Receptor Gamma

Table S2. Comparison of MN-MS^2 based on MS2DeepScore and MN-FP

tp = number of true positives, fp = number of false positives, tn = number of true negatives, fn = number of false negatives, BA = balanced accuracy

Assay	Similarity	Count_tp	Count_fp	Count_tn	Count_fn	FPR	specificity	Precision	F1	BA	ROC-AUC	Similarity threshold	Prediction threshold
**$FPR_{TPR = 0.5}$**													
AhR	MN-MS^2	224	642	1727	223	0.271	0.729	0.259	0.341	0.615	0.680	0.5	0.200
AhR	MN-FP	224	237	2130	223	0.100	0.900	0.486	0.493	0.700	0.824	0.4	0.296
AR	MN-MS^2	101	231	2745	101	0.078	0.922	0.304	0.378	0.711	0.763	0.6	0.150
AR	MN-FP	101	57	2920	102	0.019	0.981	0.639	0.560	0.739	0.765	0.4	0.452
AR.LBD	MN-MS^2	71	175	2601	72	0.063	0.937	0.289	0.365	0.717	0.794	0.4	0.115
AR.LBD	MN-FP	71	26	2707	72	0.010	0.990	0.732	0.592	0.743	0.843	0.5	0.706
Aromatase	MN-MS^2	93	679	1620	92	0.295	0.705	0.120	0.194	0.604	0.659	0.3	0.100
Aromatase	MN-FP	91	324	1933	91	0.144	0.856	0.219	0.305	0.678	0.744	0.5	0.120
ER	MN-MS^2	203	642	1622	204	0.284	0.716	0.240	0.324	0.608	0.641	0.6	0.181
ER	MN-FP	204	435	1829	204	0.192	0.808	0.319	0.390	0.654	0.696	0.4	0.200
ER.LBD	MN-MS^2	85	472	2385	85	0.165	0.835	0.153	0.234	0.667	0.723	0.5	0.088
ER.LBD	MN-FP	85	231	2624	85	0.081	0.919	0.269	0.350	0.710	0.761	0.4	0.148
PPAR.gamma	MN-MS^2	43	921	1745	44	0.345	0.655	0.045	0.082	0.574	0.589	0.5	0.040
PPAR.gamma	MN-FP	43	541	2122	44	0.203	0.797	0.074	0.128	0.646	0.703	0.4	0.048
**$FPR_{TPR = 0.9}$**													
AhR	MN-MS^2	402	1591	778	45	0.672	0.328	0.202	0.330	0.614	0.680	0.5	0.136
AhR	MN-FP	402	1106	1261	45	0.467	0.533	0.267	0.411	0.716	0.824	0.4	0.136
AR	MN-MS^2	182	2248	728	20	0.755	0.245	0.075	0.138	0.573	0.763	0.6	0.019
AR	MN-FP	183	2113	864	20	0.710	0.290	0.080	0.146	0.596	0.765	0.4	0.025
AR.LBD	MN-MS^2	129	1968	808	14	0.709	0.291	0.062	0.115	0.597	0.794	0.4	0.019
AR.LBD	MN-FP	115	913	1820	28	0.334	0.666	0.112	0.196	0.735	0.843	0.5	0.004
Aromatase	MN-MS^2	167	1697	602	18	0.738	0.262	0.090	0.163	0.582	0.659	0.3	0.057
Aromatase	MN-FP	148	1253	1004	34	0.555	0.445	0.106	0.187	0.629	0.744	0.5	0.005
ER	MN-MS^2	366	1909	355	41	0.843	0.157	0.161	0.273	0.528	0.641	0.6	0.070
ER	MN-FP	367	1824	440	41	0.806	0.194	0.168	0.282	0.547	0.696	0.4	0.083
ER.LBD	MN-MS^2	153	2200	657	17	0.770	0.230	0.065	0.121	0.565	0.723	0.5	0.028
ER.LBD	MN-FP	153	2146	709	17	0.752	0.248	0.067	0.124	0.574	0.761	0.4	0.019
PPAR.gamma	MN-MS^2	78	2234	432	9	0.838	0.162	0.034	0.065	0.529	0.589	0.5	0.011
PPAR.gamma	MN-FP	78	2103	560	9	0.790	0.210	0.036	0.069	0.553	0.703	0.4	0.002

Table S3. Selected hyperparameters and tried values of the XGBoost MS2Tox models

Assay	Model_size	N_estimators int(100...1000)	Max_depth int(10...100)	Learning_rate float(0...0.5)	Min_child_weight float(0.001...100)	Subsample float(0...1)	Colsample_bytree float(0...1)
AhR	7K	447	24	0.055	9.124	0.696	0.522
AhR	3K	498	87	0.009	0.197	0.610	0.780
AR	7K	633	54	0.028	75.805	0.454	0.848
AR	3K	810	66	0.182	0.753	0.146	0.224
AR.LBD	7K	670	48	0.071	5.131	0.898	0.482
AR.LBD	3K	892	52	0.270	7.007	0.885	0.539
Aromatase	7K	709	39	0.256	0.756	0.957	0.178
Aromatase	3K	336	94	0.013	5.789	0.850	0.993
ER	7K	102	38	0.016	8.055	0.337	0.760
ER	3K	589	77	0.460	73.121	0.692	0.161
ER.LBD	7K	136	100	0.025	1.162	0.847	0.999
ER.LBD	3K	451	38	0.484	15.316	0.815	0.123
PPAR.GAMA	7K	415	63	0.153	0.576	0.532	0.861
PPAR.GAMA	3K	510	83	0.058	32.576	0.435	0.594

Table S4. Inclusion list of environmentally relevant contaminants

Nr	Compund	Formula	[M+H]$^+$
1	10,11-Dihydro-10-Hydroxycarbamazepine	C15H14N2O2	255.1128
2	2-(Methylthio)Benzothiazole	C8H7NS2	182.0093
3	2-Aminobenzothiazole	C7H6N2S	151.0325
4	2-Hydroxy-Benzothiazole	C7H5NOS	152.0165
5	2-Methylbenzothiazole	C8H7NS	150.0372
6	5-Chlorobenzotriazole	C6H4ClN3	154.0167
7	5-Methyl-1H-Benzotriazole	C7H7N3	134.0713
8	Acephate	C4H10NO3PS	184.0192
9	Adenosine	C10H13N5O4	268.1040
10	Amitrole	C2H4N4	85.05087
11	Ampicillin	C16H19N3O4S	350.1169
12	Aspartame	C14H18N2O5	295.1289
13	Atrazine	C8H14ClN5	216.1011
14	Atrazine-2-Hydroxy	C8H15N5O	198.1349
15	Atrazine-D5	C8H14ClN5	216.1011
16	Atrazine-Desethyl	C6H10ClN5	188.0698
17	Atrazine-Desethyl-2-Hydroxy	C6H11N5O	170.1036
18	Atrazine-Desethyl-Desisopropyl	C3H4ClN5	146.0228
19	Atrazine-Desethyl-Desisopropyl-2-Hydroxy	C3H5N5O	128.0567
20	Atrazine-Desisopropyl	C5H8ClN5	174.0541
21	Atrazine-Desisopropyl-2-Hydroxy	C5H9N5O	156.088
22	Avermectin B1A	C48H72O14	873.4995
23	Benzothiazole	C7H5NS	136.0216
24	Benzotriazole	C6H5N3	120.0556
25	Benzotriazole-5-Carboxylic Acid	C7H5N3O2	164.0455
26	Butocarboxim	C7H14N2O2S	191.0849
27	Butylamine	C4H11N	74.09643
28	Caffeine	C8H10N4O2	195.0877
29	Carbamazepine	C15H12N2O	237.1022
30	Carbamazepine-10,11-Epoxide	C15H12N2O2	253.0972
31	Cefoperazone	C25H27N9O8S2	646.1497
32	Chlormequat	C5H13ClN	123.0809
33	Chlorothiazide	C7H6ClN3O4S2	295.9561
34	Chlorpyrifos	C9H11Cl3NO3PS	349.9336
35	Clarithromycin	C38H69NO13	748.4842
36	Climbazole	C15H17ClN2O2	293.1051
37	Clotrimazole	C22H17ClN2	345.1153
38	Dazomet	C5H10N2S2	163.0358
39	Dichlorvos	C4H7Cl2O4P	220.9532
40	Dimethylphthalate	C10H10O4	195.0652
41	Diphenylphthalate	C20H14O4	319.0965
42	Efavirenz	C14H9ClF3NO2	316.0347
43	Emamectin B1A	C49H75NO13	886.5311
44	Guanylurea	C2H6N4O	103.0614
45	Haloperidol	C21H23ClFNO2	376.1474
46	Histamine	C5H9N3	112.0869
47	Imazalil	C14H14Cl2N2O	297.0556
48	Irgarol	C11H19N5S	254.1434
49	Ivermectin B1A	C48H74O14	875.5151
50	Ketoconazole	C26H28Cl2N4O4	531.1560
51	L-Alanine	C3H7NO2	90.05495
52	L-Phenylalanine	C9H11NO2	166.0863
53	Melamine	C3H6N6	127.0727
54	Metazachlor	C14H16ClN3O	278.1055
55	Metformin	C4H12ClN5	166.0854

Nr	Compund	Formula	[M+H]$^+$
56	Methamidophos	C2H8NO2PS	142.0086
57	Methidathion	C6H11N2O4PS3	302.9691
58	Methomyl	C5H10N2O2S	163.0536
59	Metolachlor	C15H22ClNO2	284.1412
60	Metolachlor-Esa	C15H23NO5S	330.1370
61	Metolachlor-Oa	C15H21NO4	280.1543
62	Monuron	C9H11ClN2O	199.0633
63	Naproxen	C14H14O3	231.1016
64	Nigericin	C40H68O11	725.4834
65	Octocrylene	C24H27NO2	362.2115
66	Omethoate	C5H12NO4PS	214.0297
67	Phenazine	C12H8N2	181.0760
68	Progesterone	C21H30O2	315.2319
69	Reserpine	C33H40N2O9	609.2807
70	Rifaximin	C43H51N3O11	786.3596
71	Saccharin	C7H5NO3S	184.0063
72	Sebuthylazine	C9H16ClN5	230.1167
73	Simazine	C7H12ClN5	202.0854
74	Simazine-2-Hydroxy	C7H13N5O	184.1193
75	Simvastatin	C25H38O5	419.2792
76	Spinosad A	C41H65NO10	732.4681
77	Sucralose	C12H19Cl3O8	397.0218
78	Sudan I	C16H12N2O	249.1022
79	TCMTB	C9H6N2S3	238.9766
80	Tetraethylammonium	C8H20N	131.1669
81	Tetrahexylammonium	C24H52N	355.4173
82	Theophylline	C7H8N4O2	181.0720
83	Thiabendazole	C10H7N3S	202.0433
84	Trichlorfon	C4H8Cl3O4P	256.9299
85	Tylosin	C46H77NO17	916.5264
86	Uracil	C4H4N2O2	113.0346
87	Vancomycin	C66H75Cl2N9O24	1448.437
88	Amylamine	C5H13N	88.11208
89	2,2,2-Trifluoroacetamide	C2H2F3NO	114.0161
90	Heptylamine	C7H17N	116.1434
91	Fumeric Acid	C4H4O4	117.0182
92	Succinic Acid	C4H6O4	119.0339
93	2-Hydroxybenzonitrile	C7H5NO	120.0444
94	4-Dimethylaminopyridine	C7H10N2	123.0917
95	Dimethyl Malonate	C5H8O4	133.0495
96	2'-Aminoacetophenone	C8H9NO	136.0757
97	Tripropylamine	C9H21N	144.1747
98	Dimethyl Succinate	C6H10O4	147.0652
99	Trans-3(3-Pyridyl)Acrylic Acid	C8H7NO2	150.0550
100	N-Isopropylbenzylamine	C10H15N	150.1277
101	Vanillin	C8H8O3	153.0546
102	2,6-Dimethoxyphenol	C8H10O3	155.0703
103	4-Phenylpyridine	C11H9N	156.0808
104	1-Naphthyl Isothiocyanate	C11H7NS	186.0372
105	Tributylamine	C12H27N	186.2216
106	Ethyl Benzoylacetate	C11H12O3	193.0859
107	Dimethyl Isophthalate	C10H10O4	195.0652
108	Trans-Ferulic Acid	C10H10O4	195.0652
109	Diphenylguanidine	C13H13N3	212.1182
110	Clomazone	C12H14ClNO2	240.0786
111	Thiram	C6H12N2S4	240.9956
112	Metobromuron	C9H11BrN2O2	259.0077

Nr	Compund	Formula	[M+H]+
113	Bromacil	C9H13BrN2O2	261.0233
114	Triphenylphosphine	C18H15P	263.0984
115	Atenolol	C14H22N2O3	267.1703
116	D-(-)-Salicin	C13H18O7	287.1125
117	Iprobenfos	C13H21O3PS	289.1022
118	Chlorbromuron	C9H10BrClN2O2	292.9687
119	Tetraethylthiuram Disulfide	C10H20N2S4	297.0582
120	Tridemorph	C19H39NO	298.3104
121	Phosmet	C11H12NO4PS2	318.0018
122	Azamethiphos	C9H10ClN2O5PS	324.9809
123	Fenbuconazol	C19H17ClN4	337.1215
124	Oxyfluorfen	C15H11ClF3NO4	362.0402
125	Pyridaben	C19H25ClN2OS	365.1449
126	Tetraphenylphosphonium Chloride	C24H20ClP	375.1064
127	Nicosulfuron	C15H18N6O6S	411.1081
128	N-Benzylcinchonidinium Chloride	C26H29ClN2O	421.2041
129	Rimsulfuron	C14H17N5O7S2	432.0642
130	Fipronil	C12H4Cl2F6N4OS	436.9460
131	Penoxsulam	C16H14F5N5O5S	484.0709
132	Flufenoxuron	C21H11ClF6N2O3	489.0435
133	Lufenuron	C17H8Cl2F8N2O3	510.9857
134	Brodifacoum	C31H23BrO3	523.0903
135	Eosine Yellow (11048)	C20H6Br4Na2O5	688.6817
136	2,4,6-Trinitroaniline	C6H4N4O6	229.0204
137	2,4-Dinitroaniline	C6H5N3O4	184.0353
138	2-Methoxypyridine	C6H7NO	110.0600
139	2-Nitrobenzaldehyde	C7H5NO3	152.0342
140	3-Nitro-1H-1,2,4-Triazole	C2H2N4O2	115.0251
141	3-Nitrobenzaldehyde	C7H5NO3	152.0342
142	4-Hydroxybenzoic Acid	C7H6O3	139.0390
143	4-Methylcatechol	C7H8O2	125.0597
144	4-Nitrobenzaldehyde	C7H5NO3	152.0342
145	4-Nitrobenzoic Acid	C7H5NO4	168.0291
146	Binapacryl	C15H18N2O6	323.1238
147	Captafol	C10H9Cl4NO2S	347.9181
148	Cholesteryl Acetate	C29H48O2	429.3727
149	Ethylbenzoate	C9H10O2	151.0754
150	Fenvalerate	C25H22ClNO3	420.1361
151	Methoxychlor	C16H15Cl3O2	345.0210
152	Phorate	C7H17O2PS3	261.0201
153	Piperidine	C5H11N	86.09643
154	Pyrazophos	C14H20N3O5PS	374.0934
155	Triethylamine	C6H15N	102.1277
156	Trifluralin	C13H16F3N3O4	336.1166
157	Trimethylphosphine	C3H9P	77.05146
158	Xylose	C5H10O5	151.0601
159	Isatin	C8H5NO2	148.0393
160	Ethyl Azinphos	C12H16N3O3PS2	346.0444
161	Tepraloxydim	C17H24ClNO4	342.1467
162	Bromoxynil	C7H3Br2NO	275.8654
163	MCPA	C9H9ClO3	201.0313
164	Phenytoin	C15H12N2O2	253.0972
165	Flamprop	C16H13ClFNO3	322.0641
166	Benodanil	C13H10INO	323.9880
167	Dinoterb	C10H12N2O5	241.0819
168	Coumaphos	C14H16ClO5PS	363.0217
169	Benzoic Acid	C7H6O2	123.0441

Nr	Compund	Formula	[M+H]$^{+}$
170	Inabenfide	C19H15ClN2O2	339.0895
171	Salinomycin	C42H70O11	751.4991
172	Methylparaben	C8H8O3	153.0546
173	Salicylic Acid	C7H6O3	139.0390
174	Triclosan	C12H7Cl3O2	288.9584
175	Linuron	C9H10Cl2N2O2	249.0192
176	Valproic Acid	C8H16O2	145.1223

Table S5. Applied MS-DIAL parameters

**Data collection**	
MS1 tolerance	0.001 Da
MS2 tolerance	0.003 Da
RT begin	2 min
RT end	25 min
MS1 mass range begin	50 Da
MS1 mass range end	1050 Da
MS/MS mass range begin	50 Da
MS/MS mass range end	1050 Da
Maximum charged number	2
Consider Cl and Br elements	Yes
**Peak detection**	
Min peak height	10000 amplitude
Mass slice width	0.07 Da
Smoothing method	Linear weighted moving average
Smoothing level	5 scan
Min peak width	8 scan
**MS2Dec**	
Sigma window value	1
MS/MS abundance cut off	0 amplitude
Exclude after precursor ion	Yes
Keep the isotopic ions until	5 Da
Keep the isotopic ions w/o MS2Dec	Yes
**Identification (default settings)**	
MSP file:	None
RT tolerance	100 min
MS1 accurate mass tolerance	0.01 Da
MS2 accurate mass tolerance	0.05 Da
Identification score cut off	80 %
Use RT for scoring	No
Use RT for filtering	No
Post identification	None
RT tolerance	0.2 min
Accurate mass tolerance	0.002 Da
Identification score cut off	85 %
Rel. abundance cut off	0 %
Only report the top hit	No

**Adduct**	
Negative ionization mode	[M-H]-
Positive ionization mode	[M+H]+,
**Alignment**	
RT tolerance	0.75 min
MS1 tolerance	0.003 Da
RT factor	0.5
MS1 factor	0.5
N% detected in at least one group	100 %
Remove features based on blank information	Sample max/blank average
Fold change	10
Keep ref.matched features	Yes
keep suggested w/o MS2 features	No
Keep removable features and assign the tag	Yes
Gap filling by compulsion	Yes

Table S6. Number of available compound labels for each assay in the test set (N = 861)

Assay	Inconclusives	Inactives	Actives	Usable compounds	Percent actives
AhR	163	590	108	698	15.5%
AR	92	734	35	769	4.6%
AR.LBD	145	696	20	716	2.8%
Aromatase	249	584	28	612	4.6%
ER	200	582	79	661	12.0%
ER.LBD	118	716	27	743	3.6%
PPAR.gamma	178	666	17	683	2.5%

Table S7. Spearman correlation

Spearman correlation test results between the three evaluated spectral similarities with the Tanimoto similarity. The S statistic is a part of the test and represents the sum of squared rank differences. It's part of the calculation to determine the correlation coefficient. The exact value here is generally not interpreted directly but is used in the background calculation.

Parameter	Greedy cosine	Modified cosine	MS2DeepScore
S	2.7654e+19	2.8458e+19	2.0247e+19
p-value	< 2.2e-16	< 2.2e-16	< 2.2e-16
**rho**	**0.159**	**0.135**	**0.385**

Table S8. MN-MS^2 test set results

Assay	Metrics set	TP	FP	TN	FN	TPR	FPR	Precision	F1	BA	ROC-AUC	Prediction threshold
AhR	$FPR_{TPR=0.5}$	54	132	458	54	0.500	0.224	0.290	0.367	0.638	0.705	0.201
AR	$FPR_{TPR=0.5}$	18	107	625	17	0.514	0.146	0.144	0.225	0.684	0.769	0.080
AR.LBD	$FPR_{TPR=0.5}$	10	28	668	10	0.500	0.040	0.263	0.345	0.730	0.865	0.128
Aromatase	$FPR_{TPR=0.5}$	14	172	412	14	0.500	0.295	0.075	0.131	0.603	0.643	0.092
ER	$FPR_{TPR=0.5}$	40	223	358	39	0.506	0.384	0.152	0.234	0.561	0.577	0.142
ER.LBD	$FPR_{TPR=0.5}$	14	167	549	13	0.519	0.233	0.077	0.135	0.643	0.672	0.065
PPAR.gamma	$FPR_{TPR=0.5}$	9	259	407	8	0.529	0.389	0.034	0.063	0.570	0.620	0.032
AhR	$FPR_{TPR=0.9}$	97	486	104	11	0.898	0.824	0.166	0.281	0.537	0.705	0.052
AR	$FPR_{TPR=0.9}$	30	575	157	5	0.857	0.786	0.050	0.094	0.536	0.769	0.003
AR.LBD	$FPR_{TPR=0.9}$	18	289	407	2	0.900	0.415	0.059	0.110	0.742	0.865	0.024
Aromatase	$FPR_{TPR=0.9}$	25	392	192	3	0.893	0.671	0.060	0.112	0.611	0.643	0.039
ER	$FPR_{TPR=0.9}$	71	520	61	8	0.899	0.895	0.120	0.212	0.502	0.577	0.034
ER.LBD	$FPR_{TPR=0.9}$	24	581	135	3	0.889	0.811	0.040	0.076	0.539	0.672	0.018
PPAR.gamma	$FPR_{TPR=0.9}$	15	594	72	2	0.882	0.892	0.025	0.048	0.495	0.620	0.002

Table S9. CP test results. Bold rows indicate the best significance and CoverageP per endpoint

Assay	Sign. level	Precision	F1	ROC-AUC no both empty	FPR	TPR	$FPR_{TPR=0.9}$	$FPR_{TPR=0.5}$	MCC	BA	CoverageP	CoverageN
AhR	0.10	0.289	0.375	0.713	0.143	0.536	0.738	0.143	0.303	0.696	0.259	0.439
AhR	0.15	0.337	0.441	0.742	0.187	0.638	0.699	0.146	0.352	0.726	0.435	0.536
AhR	0.20	0.333	0.447	0.744	0.218	0.678	0.725	0.161	0.353	0.730	0.546	0.622
AhR	0.25	0.312	0.427	0.739	0.253	0.676	0.711	0.153	0.321	0.712	0.657	0.710
**AhR**	**0.30**	**0.295**	**0.412**	**0.729**	**0.283**	**0.679**	**0.648**	**0.190**	**0.297**	**0.698**	**0.778**	**0.814**
AR.LBD	0.10	0.171	0.289	0.926	0.310	0.923	0.011	0.000	0.317	0.807	0.650	0.269
AR.LBD	0.15	0.125	0.220	0.916	0.256	0.929	0.206	0.000	0.286	0.836	0.700	0.510
AR.LBD	0.20	0.106	0.189	0.869	0.264	0.833	0.505	0.000	0.236	0.785	0.900	0.685
**AR.LBD**	**0.25**	**0.091**	**0.165**	**0.854**	**0.281**	**0.850**	**0.409**	**0.000**	**0.219**	**0.785**	**1.000**	**0.865**
AR.LBD	0.30	0.089	0.162	0.857	0.249	0.842	0.640	0.000	0.222	0.797	0.950	0.943
Aromatase	0.10	0.125	0.217	0.873	0.156	0.833	0.661	0.018	0.285	0.839	0.214	0.384
Aromatase	0.15	0.130	0.220	0.829	0.220	0.714	0.336	0.115	0.237	0.747	0.500	0.521
Aromatase	0.20	0.104	0.182	0.807	0.250	0.733	0.468	0.092	0.209	0.742	0.536	0.651
Aromatase	0.25	0.096	0.169	0.788	0.287	0.722	0.528	0.117	0.186	0.717	0.643	0.733
**Aromatase**	**0.30**	**0.098**	**0.172**	**0.760**	**0.308**	**0.696**	**0.548**	**0.211**	**0.173**	**0.694**	**0.821**	**0.819**
AR	0.10	0.250	0.395	0.934	0.402	0.944	0.370	0.000	0.359	0.771	0.514	0.173
AR	0.15	0.162	0.270	0.887	0.315	0.810	0.527	0.000	0.264	0.747	0.600	0.380
AR	0.20	0.136	0.232	0.839	0.297	0.792	0.677	0.000	0.242	0.748	0.686	0.556
AR	0.25	0.119	0.206	0.810	0.297	0.750	0.747	0.004	0.214	0.727	0.800	0.711
**AR**	**0.30**	**0.108**	**0.188**	**0.786**	**0.298**	**0.719**	**0.701**	**0.071**	**0.193**	**0.711**	**0.914**	**0.869**
ER.LBD	0.10	0.131	0.219	0.738	0.305	0.667	0.724	0.190	0.190	0.681	0.444	0.243
ER.LBD	0.15	0.081	0.142	0.701	0.266	0.571	0.860	0.114	0.133	0.653	0.519	0.478
ER.LBD	0.20	0.065	0.115	0.678	0.258	0.500	0.892	0.097	0.100	0.621	0.593	0.622
ER.LBD	0.25	0.057	0.103	0.652	0.265	0.500	0.914	0.234	0.092	0.618	0.667	0.781
**ER.LBD**	**0.30**	**0.053**	**0.095**	**0.639**	**0.281**	**0.476**	**0.850**	**0.335**	**0.076**	**0.598**	**0.778**	**0.895**
ER	0.10	0.211	0.320	0.712	0.319	0.667	0.894	0.177	0.230	0.674	0.228	0.242
ER	0.15	0.146	0.229	0.683	0.342	0.522	0.732	0.195	0.113	0.590	0.291	0.352
ER	0.20	0.182	0.272	0.650	0.346	0.541	0.785	0.342	0.133	0.597	0.468	0.447

Assay	Sign. level	Precision	F1	ROC-AUC no both empty	FPR	TPR	$FPR_{TPR=0.9}$	$FPR_{TPR=0.5}$	MCC	BA	CoverageP	CoverageN
ER	0.25	0.174	0.265	0.633	0.355	0.558	0.791	0.312	0.135	0.602	0.544	0.552
**ER**	**0.30**	**0.175**	**0.261**	**0.621**	**0.343**	**0.518**	**0.745**	**0.338**	**0.120**	**0.588**	**0.709**	**0.687**
PPAR.gamma	0.10	0.059	0.111	0.634	0.787	1.000	0.705	0.344	0.112	0.607	0.177	0.092
PPAR.gamma	0.15	0.059	0.111	0.768	0.506	1.000	0.437	0.278	0.170	0.747	0.294	0.237
PPAR.gamma	0.20	0.061	0.112	0.642	0.429	0.700	0.591	0.282	0.105	0.636	0.588	0.378
PPAR.gamma	0.25	0.054	0.100	0.649	0.414	0.667	0.681	0.275	0.093	0.626	0.706	0.508
**PPAR.gamma**	**0.30**	**0.044**	**0.082**	**0.655**	**0.409**	**0.667**	**0.748**	**0.217**	**0.085**	**0.629**	**0.706**	**0.643**

Table S10. MS2Tox models test set performance without constraints

Assay	Training set	ROC-AUC	FPR	TPR	BA	Precision	F1
AhR	3K	0.860	0.031	0.435	0.702	0.723	0.543
AR	3K	0.762	0.011	0.486	0.737	0.680	0.567
AR.LBD	3K	0.822	0.010	0.700	0.845	0.667	0.683
Aromatase	3K	0.852	0.002	0.107	0.553	0.750	0.188
ER	3K	0.698	0.021	0.101	0.540	0.400	0.162
ER.LBD	3K	0.788	0.011	0.296	0.643	0.500	0.372
PPAR.gamma	3K	0.732	0.000	0.000	0.500	0.000	0.000
AhR	7K	0.873	0.034	0.417	0.691	0.692	0.520
AR	7K	0.804	0.000	0.000	0.500	0.000	0.000
AR.LBD	7K	0.893	0.003	0.700	0.849	0.875	0.778
Aromatase	7K	0.861	0.014	0.214	0.600	0.429	0.286
ER	7K	0.680	0.003	0.089	0.543	0.778	0.159
ER.LBD	7K	0.787	0.001	0.296	0.647	0.889	0.444
PPAR.gamma	7K	0.890	0.003	0.118	0.557	0.500	0.190

Table S11. MS2Tox models test set performance at threshold resulting in TPR=0.5

Assay	Training set	ROC-AUC	FPR	Threshold	BA	Precision	F1
AhR	3K	0.860	0.039	0.374	0.731	0.701	0.584
AR	3K	0.762	0.042	0.146	0.736	0.367	0.429
AR.LBD	3K	0.822	0.000	0.922	0.750	1.000	0.667
Aromatase	3K	0.852	0.094	0.119	0.703	0.203	0.289
ER	3K	0.698	0.204	0.186	0.651	0.252	0.336
ER.LBD	3K	0.788	0.066	0.157	0.726	0.230	0.318
PPAR.gamma	3K	0.732	0.201	0.036	0.664	0.063	0.113
AhR	7K	0.873	0.054	0.360	0.723	0.628	0.557
AR	7K	0.804	0.093	0.064	0.711	0.209	0.298
AR.LBD	7K	0.893	0.000	0.926	0.750	1.000	0.667
Aromatase	7K	0.861	0.045	0.025	0.728	0.350	0.412
ER	7K	0.680	0.196	0.149	0.655	0.260	0.343
ER.LBD	7K	0.787	0.098	0.061	0.710	0.167	0.252
PPAR.gamma	7K	0.890	0.033	0.022	0.748	0.290	0.375

Table S12. MS2Tox models test set performance at threshold resulting in TPR=0.9.

Assay	Training set	ROC-AUC	FPR	Threshold	BA	Precision	F1
AhR	3K	0.860	0.375	0.033	0.766	0.307	0.459
AR	3K	0.762	0.770	0.000	0.572	0.054	0.101
AR.LBD	3K	0.822	0.718	0.000	0.591	0.035	0.067
Aromatase	3K	0.852	0.322	0.038	0.803	0.121	0.215
ER	3K	0.698	0.785	0.050	0.563	0.136	0.237
ER.LBD	3K	0.788	0.764	0.002	0.581	0.044	0.083
PPAR.gamma	3K	0.732	0.635	0.029	0.653	0.036	0.070
AhR	7K	0.873	0.349	0.042	0.779	0.322	0.476
AR	7K	0.804	0.729	0.028	0.593	0.056	0.106
AR.LBD	7K	0.893	0.478	0.001	0.711	0.051	0.097
Aromatase	7K	0.861	0.440	0.000	0.744	0.092	0.167
ER	7K	0.680	0.754	0.095	0.579	0.141	0.244
ER.LBD	7K	0.787	0.735	0.012	0.596	0.045	0.087
PPAR.gamma	7K	0.890	0.518	0.000	0.712	0.044	0.085

Table S13. SHAP analysis of the MS2Tox AhR model

Fingerprint feature	SMARTS	Description	Feature importance
absoluteIndex_785	>= 2 aromatic rings	2 or more aromatic rings	0.246
absoluteIndex_8469	c(:c:c:c:c:1):c:1	Six-membered aromatic ring composed exclusively of carbon atoms	0.221
absoluteIndex_921	[#7&!H0](~[#6])(N(~C)(~H))	Nitrogen atom connected to at least one hydrogen atom and to a carbon atom (any bond type)	0.192
absoluteIndex_142	[$([#6X3H0][#6]),$([#6X3H])](=[!#6])[!#6]	Carbon with three bonds, excluding hydrogen, with one single bond to a carbon atom and one single and one double bond to non-carbon atoms	0.168
absoluteIndex_497	[#8]=* >= 2	At least two oxygen atoms double-bonded to any atom	0.143
absoluteIndex_473	*~*(~*)(~*)~*	Any atom with four neighbouring atoms (any type)	0.142
absoluteIndex_354	[$([#7X2,OX1,SX1]=*[!H0;!$([a;!n])]),$([#7X3,OX2,SX2;!H0]*=*),$([#7X3,OX2,SX2;!H0]*:n)]	Nitrogen with two bonds, or oxygen or sulphur with one bond, attached by a double bond to any atom that has at least one hydrogen and is not connected to an aromatic atom other than nitrogen; or nitrogen with three bonds, or oxygen or sulphur with two bonds, bearing at least one hydrogen and attached to any atom that is either double-bonded to another atom (any type) or single-bonded to an aromatic nitrogen	0.142
absoluteIndex_4632	CC	Two aliphatic carbon atoms connected by a single	0.139
absoluteIndex_27	C[OH]	Carbon atom connected with hydroxyl group	0.136
absoluteIndex_8524	c(:c:c:c:c:1):c:1~[#7]	Six-membered aromatic ring composed exclusively of carbon atoms, connected with a nitrogen atom (any bond type)	0.129

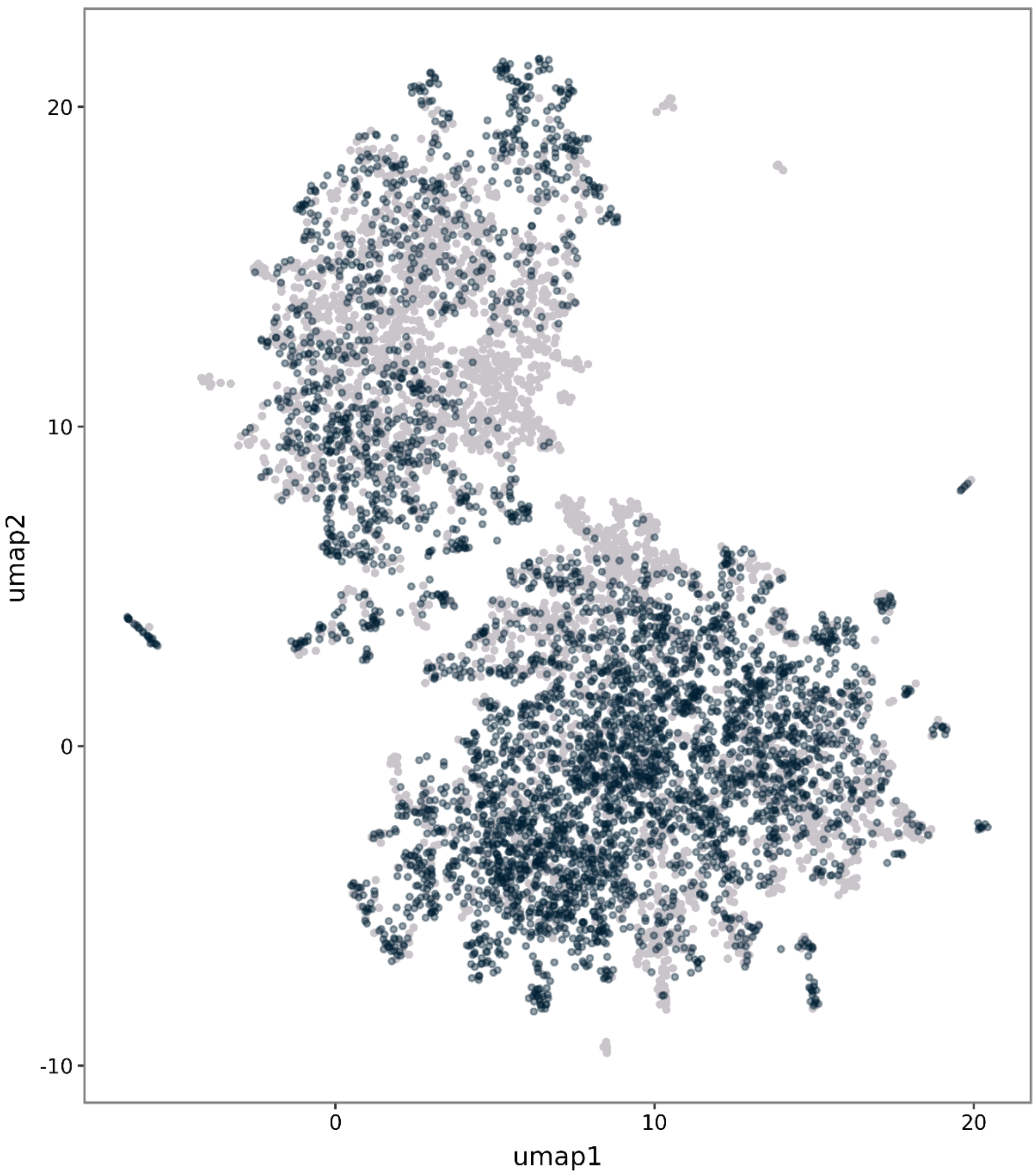

Fig. S1 Chemical space visualization with UMAP
(n_neighbourse = 10, min_dist = 0.8, euclidean distance score) on compounds of the original dataset (grey) and compounds of the MS^2 dataset (dark blue).

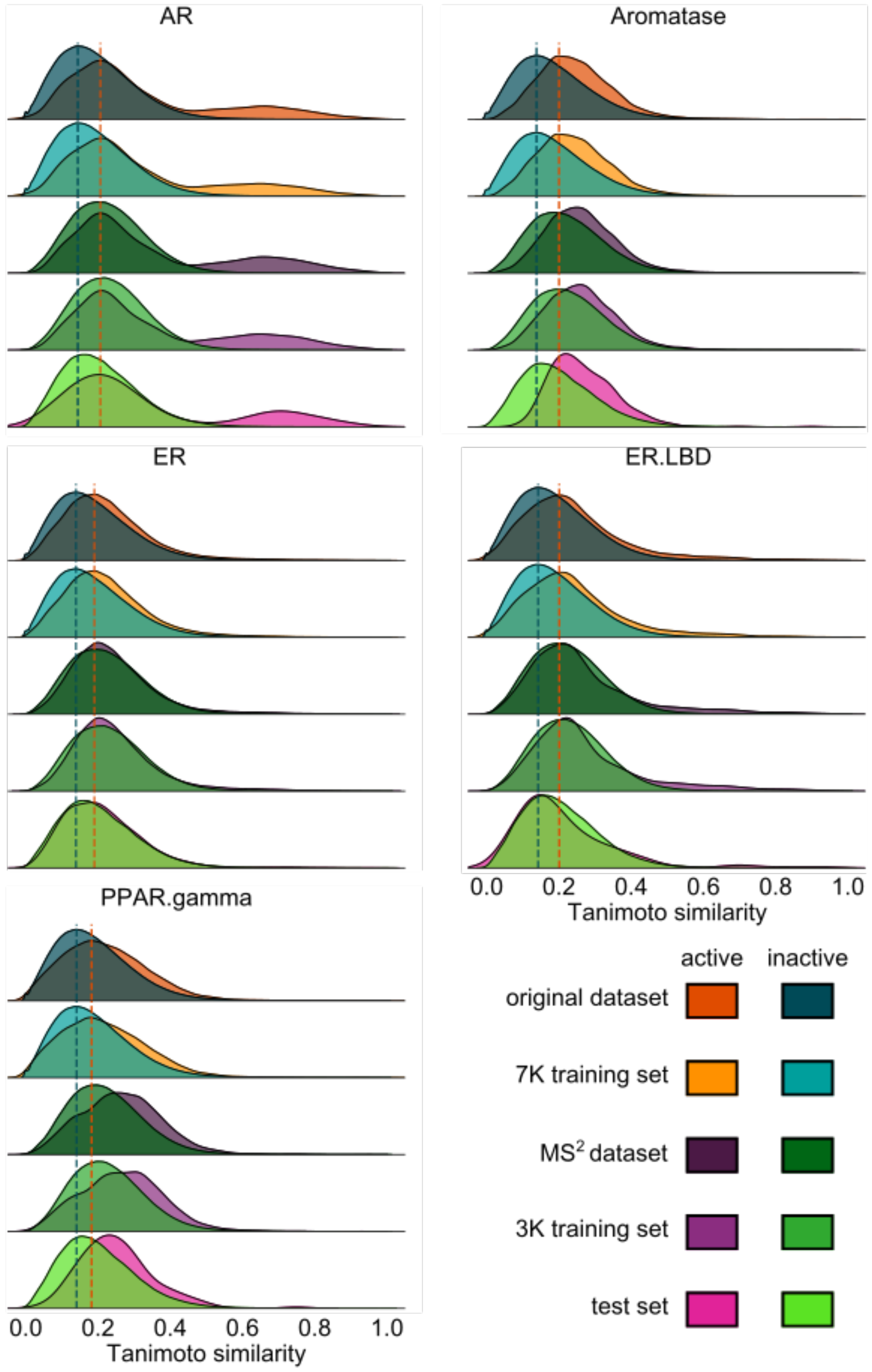

Fig. S2 Tanimoto similarity distribution

Similarity distribution of active-to-active and inactive-to-active compounds calculated from SIRIUS+CSI:FingerID (ver. 5.6.3) fingerprints respectively are shown for each used dataset. The dotted line represents the maximum of inactives (blue) and actives (orange) in the original dataset.

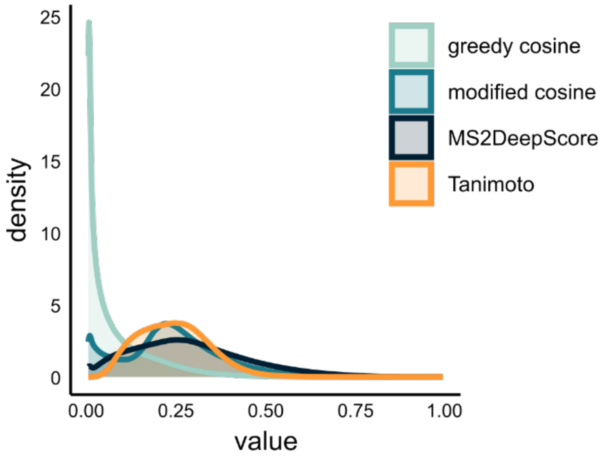

Fig. S3 Distribution of calculated similarities between spectra pairs of each calculated similarity type

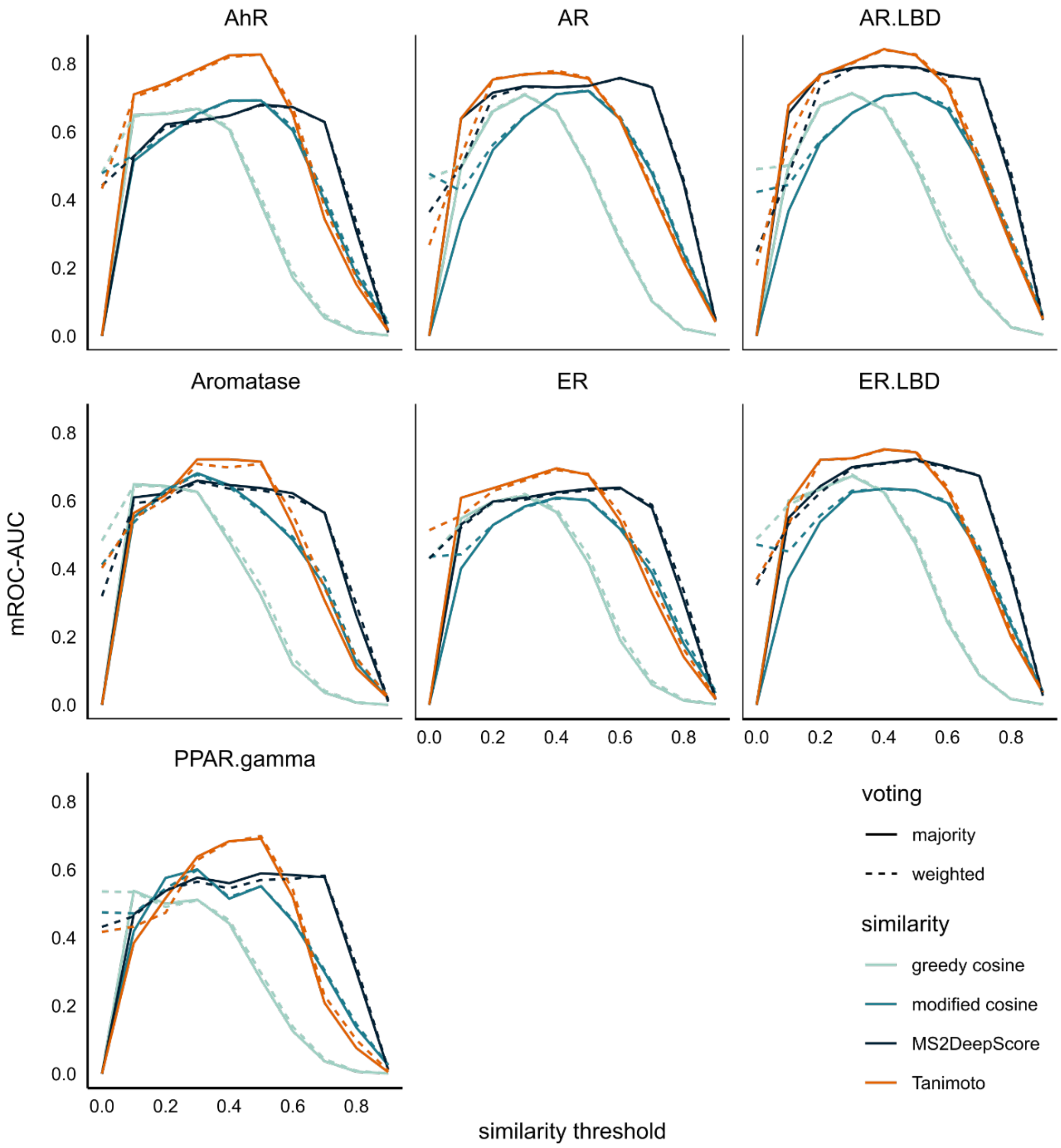

Fig. S4 mROC-AUC values for MN activity annotation for all hyperparameter combinations of all assays

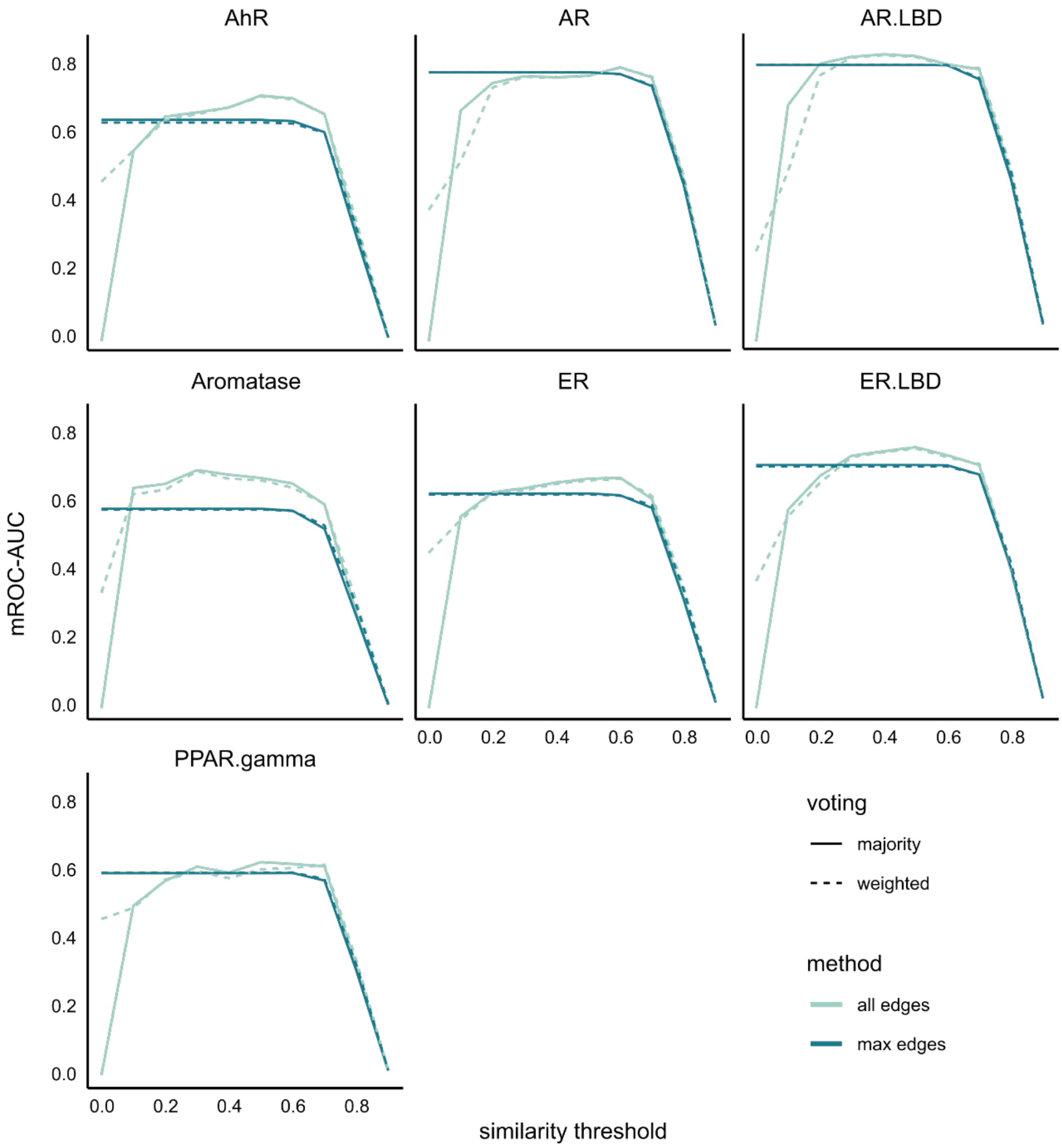

Fig. S5 mROC-AUC values for activity annotation on MS2DeepScore MN-MS^2
Comparison between taking into account all edges or a maximum of edges (n=6).

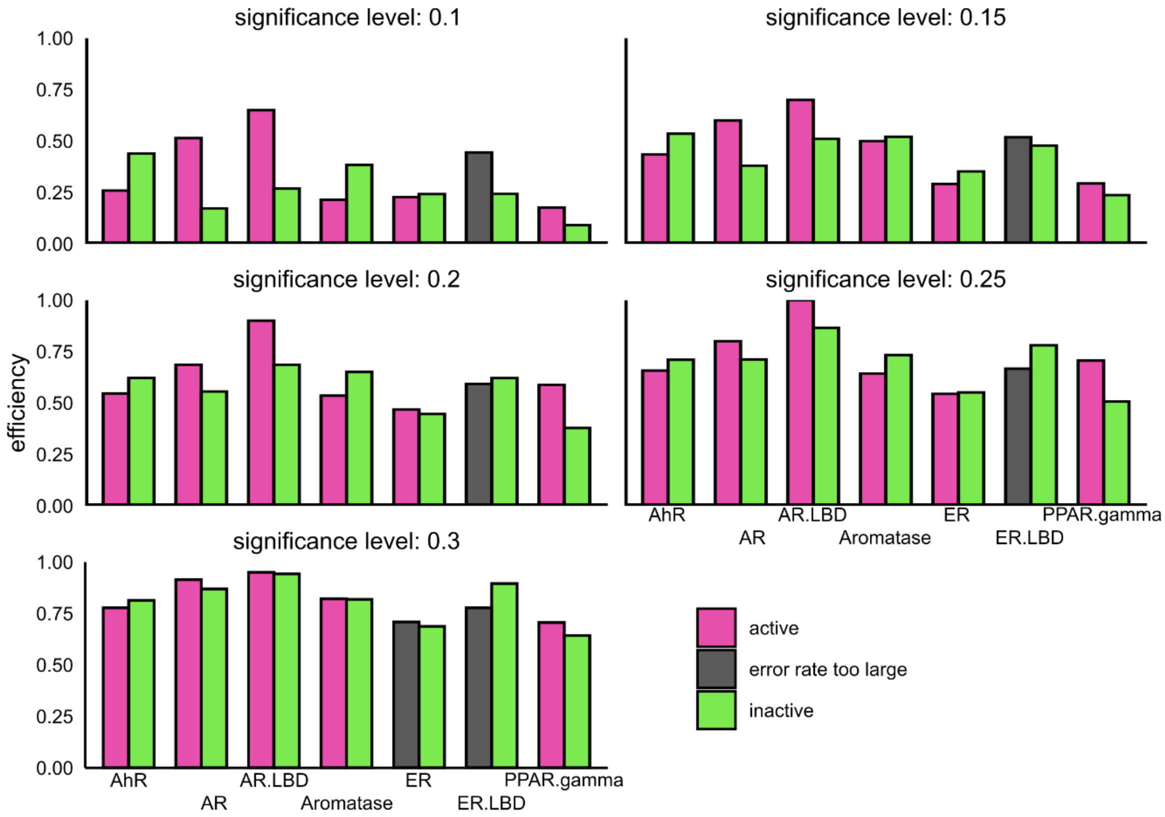

Fig. S6 Efficiencies of the investigated test data sets at various significance levels

Significance levels correspond to acceptable error rates. Examples where the error rate is too large are indicated by grey bars.

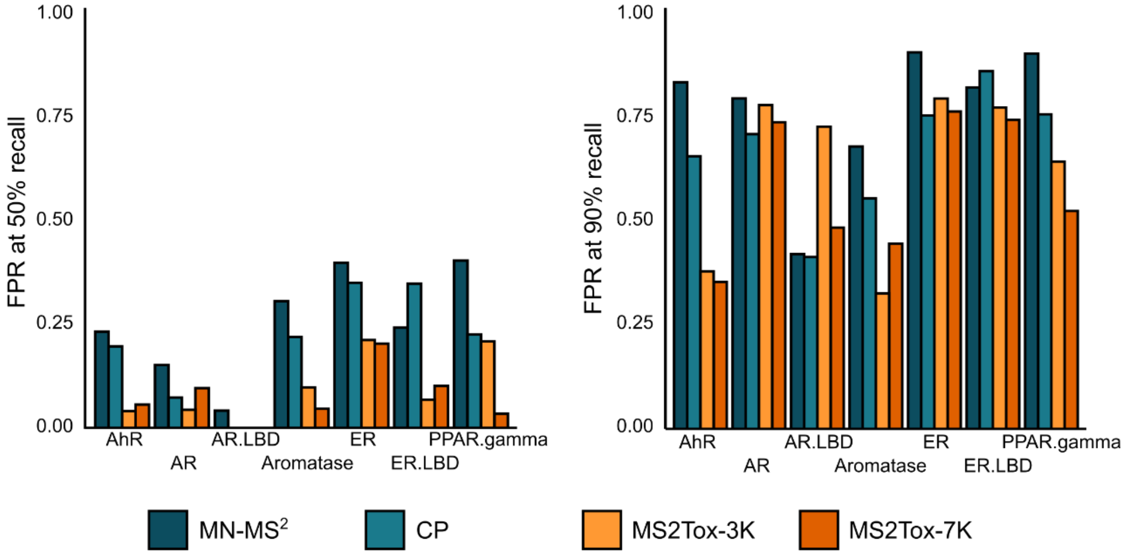

Fig. S7 Comparison of FPR at 50% recall and at 90% recall

Comparison between approaches for all nuclear receptor endpoints on the test set (n = 861, number of actives in test set are 108 for AhR, 35 for AR, 20 for AR.LBD, 28 for Aromatase, 79 for ER, 27 for ER.LBD and 17 for PPAR.gamma).

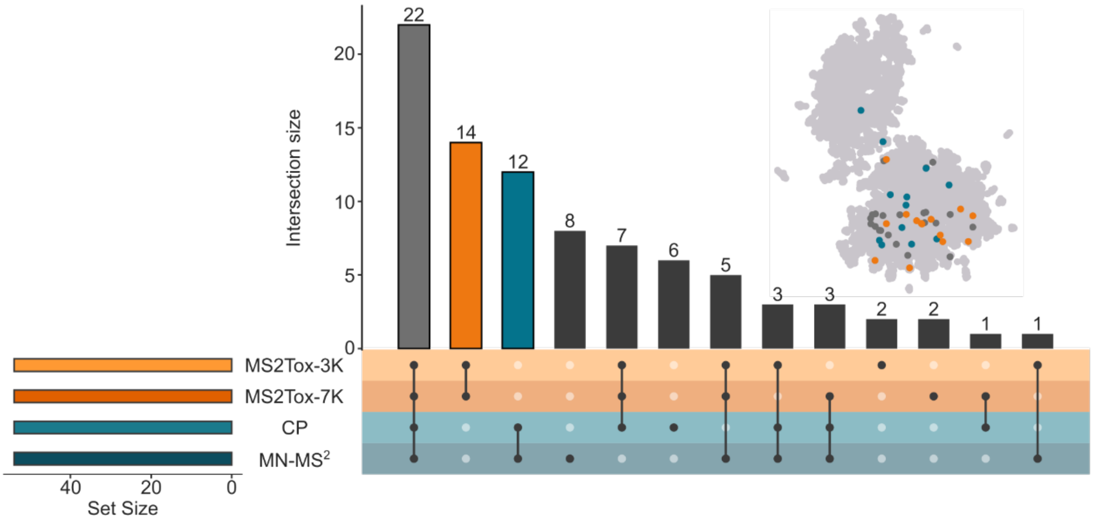

Fig. S8 UpSet plot of overlapping true positive predictions in the test set for AhR at 50% recall

Next to it is a chemical space UMAP representation of the original dataset in light grey. Highlighted are 22 true positive compounds in dark grey, that are labeled by all approaches, in orange only true positives labeled compounds by fingerprint-based models, and in blue only true positive compounds labeled by approaches based on MS^2 similarity.

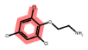

431 - (1) MassBank

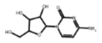

431 - (2) MN

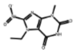

431 - (3) SIRIUS structure

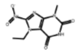

431 - (4) SIRIUS formula

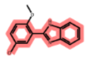

458 - (1) MassBank

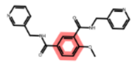

458 - (2) MN

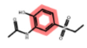

458 - (3) SIRIUS structure

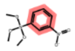

458 - (4) SIRIUS formula

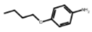

325 - (2) MN

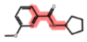

325 - (3) SIRIUS structure

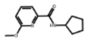

325 - (4) SIRIUS formula

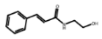

415 - (2) MN

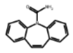

415 - (3) SIRIUS structure

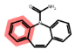

415 - (4) SIRIUS formula

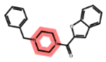

453 - (2) MN

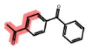

453 - (3) SIRIUS structure

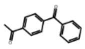

453 - (4) SIRIUS formula

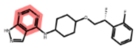

509 - (2) MN

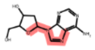

509 - (3) SIRIUS structure

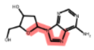

509 - (4) SIRIUS formula

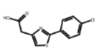

515 - (2) MN

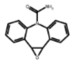

515 - (3) SIRIUS structure

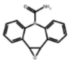

515 - (4) SIRIUS formula

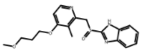

516 - (2) MN

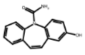

516 - (3) SIRIUS structure

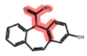

516 - (4) SIRIUS formula

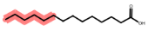

532 - (2) MN

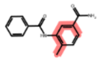

532 - (3) SIRIUS structure

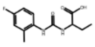

532 - (4) SIRIUS formula

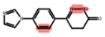

656 - (2) MN

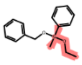

656 - (3) SIRIUS structure

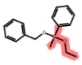

656 - (4) SIRIUS formula

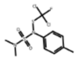

682 - (2) MN

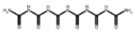

682 - (3) SIRIUS structure

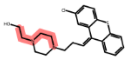

935 - (2) MN

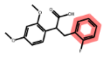

935 - (3) SIRIUS structure

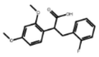

935 - (4) SIRIUS formula

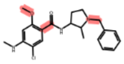

965 - (2) MN

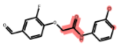

965 - (3) SIRIUS structure

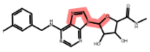

989 - (2) MN

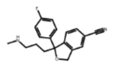

989 - (3) SIRIUS structure

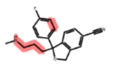

989 - (4) SIRIUS formula

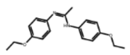

1033 - (2) MN

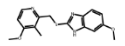

1033 - (3) SIRIUS structure

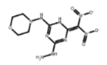

1033 - (4) SIRIUS formula

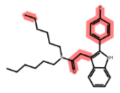

1306 - (2) MN

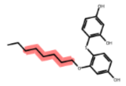

1306 - (3) SIRIUS structure

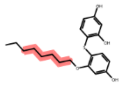

1306 - (4) SIRIUS formula

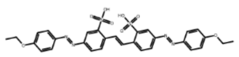

1405 - (2) MN

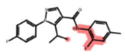

1405 - (3) SIRIUS structure

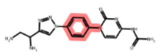

1405 - (4) SIRIUS formula

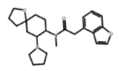

1453 - (2) MN

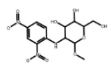

1453 - (3) SIRIUS structure

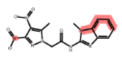

1453 - (4) SIRIUS formula

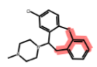

1593 - (2) MN

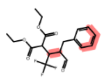

1593 - (3) SIRIUS structure

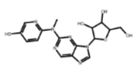

1593 - (4) SIRIUS formula

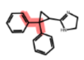

1816 - (2) MN

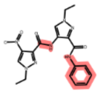

1816 - (3) SIRIUS structure

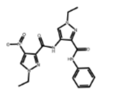

1816 - (4) SIRIUS formula

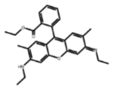

1959 - (2) MN

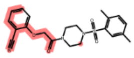

1959 - (3) SIRIUS structure

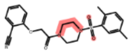

1959 - (4) SIRIUS formula

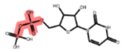

3000 - (2) MN

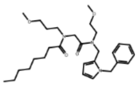

3000 - (3) SIRIUS structure

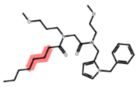

3000 - (4) SIRIUS formula

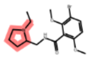

4171 - (2) MN

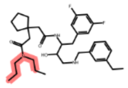

4171 - (3) SIRIUS structure

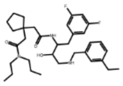

4171 - (4) SIRIUS formula

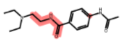

4172 - (2) MN

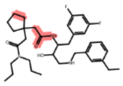

4172 - (3) SIRIUS structure

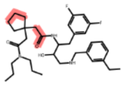

4172 - (4) SIRIUS formula

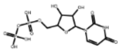

4610 - (2) MN

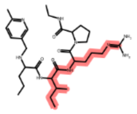

4610 - (3) SIRIUS structure

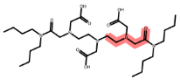

4610 - (4) SIRIUS formula

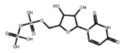

4967 - (2) MN

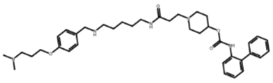

4967 - (3) SIRIUS structure

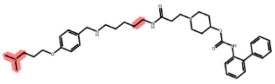

4967 - (4) SIRIUS formula

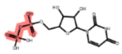

5474 - (2) MN

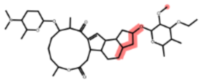

5474 - (3) SIRIUS structure

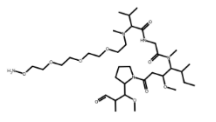

5474 - (4) SIRIUS formula

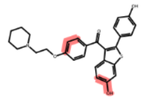

5922 - (2) MN

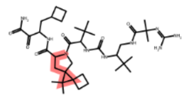

5922 - (3) SIRIUS structure

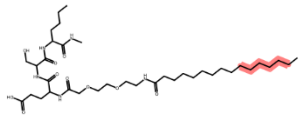

5922 - (4) SIRIUS formula

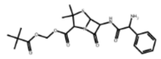

6025 - (2) MN

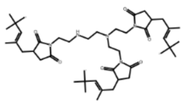

6025 - (3) SIRIUS structure

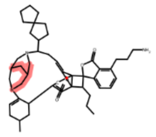

6025 - (4) SIRIUS formula

Fig. S9 Candidate structures obtained using four complementary methods: (1) library matching against MassBank spectra using cosine similarity threshold of 0.7 and requiring at least four matching fragment peaks; (2) querying the compound from the *MS² dataset* (MassBank, MoNA, NIST overlap with Tox21) with the highest MS2DeepScore for the detected LC/HRMS feature; (3) retrieving the top-ranked candidate structure from SIRIUS+CSI:FingerID; and (4) retrieving the top-ranked candidate structure associated with the highest-ranked molecular formula in SIRIUS+CSI:FingerID. Scaffolds associated with potential endocrine-disrupting activity are highlighted.

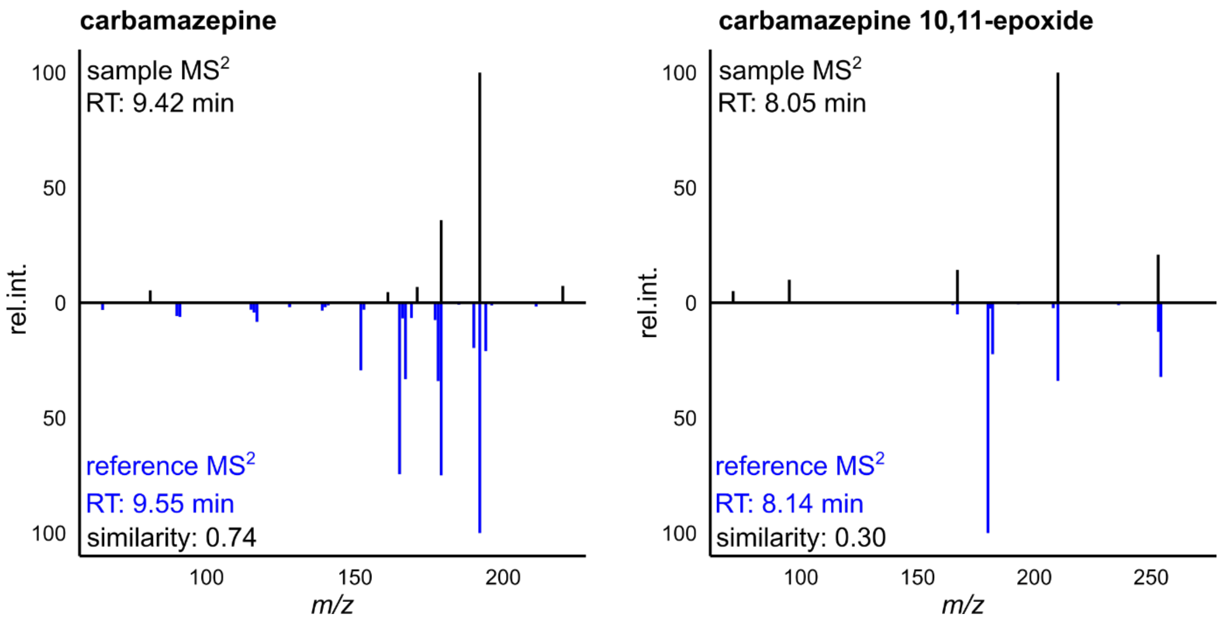

Fig. S10 Carbamazepine and carbamazepine 10,11-epoxide spectra comparison to spectral references.

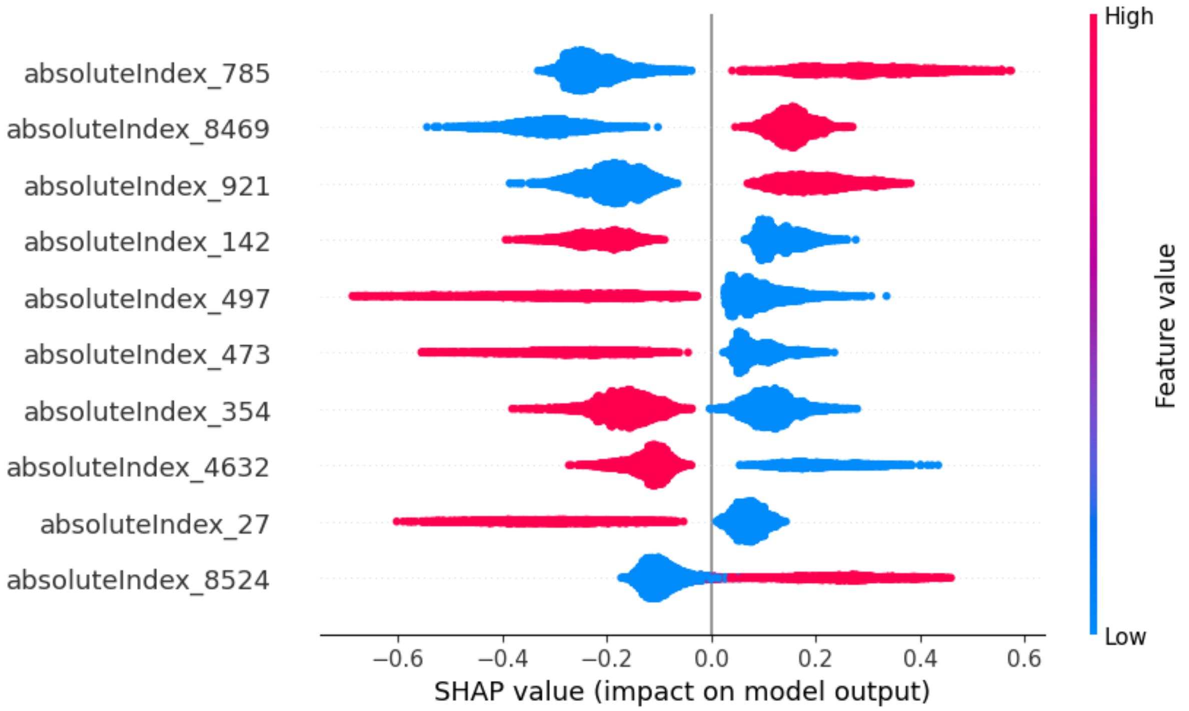

Fig. S11 Variable importance analysis of the MS2Tox AhR activity prediction model

High (red, value = 1) indicates that the feature is present, and low (blue, value = 0) indicates the absence of a feature. Interpretation of each feature can be found in Table S13.

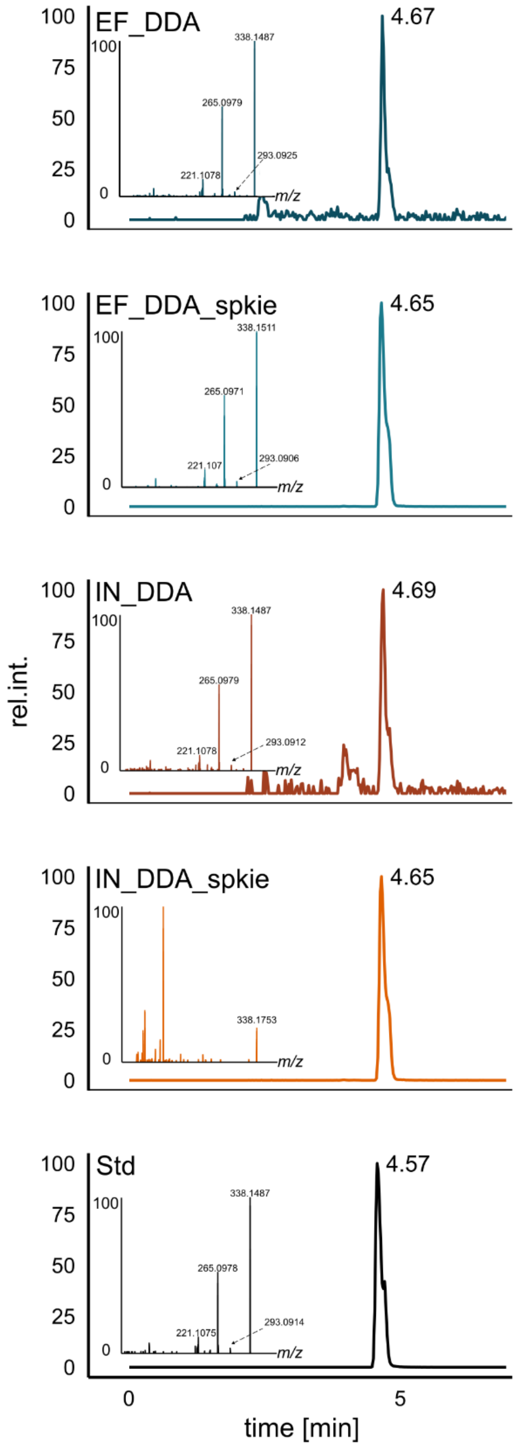

Fig. S12 Confirmation of Zolpidem phenyl-4-carboxylic acid

Characteristic transitions are *m/z* 338.2 to *m/z* 265.2 and *m/z* 338.2 to *m/z* 293.1.[7] Zolpidem phenyl-4-carboxylic-acid has been identified in the effluent sample (EF) and the influent sample (IN).

References

(1) Papenberg, M.; Klau, G. W. Using Anticlustering to Partition Data Sets into Equivalent Parts. *Psychological Methods* **2021**, *26* (2), 161–174. https://doi.org/10.1037/met0000301.

(2) Papenberg, M.; algorithm), M. M. (centroid based clustering; Klau, G. W.; logo), J. V. N. (package; al.), M. B. (Bicriterion algorithm by B. et; set), M. L. S. (Example data; algorithm), M. D. (Optimal maximum dispersion. Anticlust: Subset Partitioning via Anticlustering, 2024. https://cran.r-project.org/web/packages/anticlust/index.html (accessed 2024-06-18).

(3) McInnes, L.; Healy, J.; Melville, J. UMAP: Uniform Manifold Approximation and Projection for Dimension Reduction. arXiv September 17, 2020. https://doi.org/10.48550/arXiv.1802.03426.

(4) Pedregosa, F.; Varoquaux, G.; Gramfort, A.; Michel, V.; Thirion, B.; Grisel, O.; Blondel, M.; Prettenhofer, P.; Weiss, R.; Dubourg, V.; Vanderplas, J.; Passos, A.; Cournapeau, D.; Brucher, M.; Perrot, M.; Duchesnay, É. Scikit-Learn: Machine Learning in Python. *Journal of Machine Learning Research* **2011**, *12* (85), 2825–2830.

(5) Norinder, U.; Myatt, G.; Ahlberg, E. Predicting Aromatic Amine Mutagenicity with Confidence: A Case Study Using Conformal Prediction. *Biomolecules* **2018**, *8* (3), 85. https://doi.org/10.3390/biom8030085.

(6) Lundqvist, J.; Lavonen, E.; Mandava, G.; Selin, E.; Ejhed, H.; Oskarsson, A. Effect-Based Monitoring of Chemical Hazards in Drinking Water from Source to Tap: Seasonal Trends over 2 Years of Sampling. *Environ Sci Eur* **2024**, *36* (1), 45. https://doi.org/10.1186/s12302-024-00875-z.

(7) Feng, X.; Xiang, P.; Chen, H.; Shen, M. LC–MS-MS with Post-Column Reagent Addition for the Determination of Zolpidem and Its Metabolite Zolpidem Phenyl-4-Carboxylic Acid in Oral Fluid after a Single Dose. *Journal of Analytical Toxicology* **2017**, *41* (9), 735–743. https://doi.org/10.1093/jat/bkx062.
